# Exercise‐Activated mPFC Tri‐Synaptic Pathway Ameliorates Depression‐Like Behaviors in Mouse

**DOI:** 10.1002/advs.202408618

**Published:** 2024-11-22

**Authors:** Tian Lan, Ye Li, Xiao Chen, Wenjing Wang, Changmin Wang, Haiyan Lou, Shihong Chen, Shuyan Yu

**Affiliations:** ^1^ Shandong Key Laboratory of Mental Disorders and Intelligent Control The Second Hospital of Shandong University School of Basic Medical Sciences Shandong University Jinan Shandong 250012 China; ^2^ Department of Physiology School of Basic Medical Sciences Cheeloo College of Medicine Shandong University Jinan Shandong 250012 China; ^3^ Department of Pharmacology School of Basic Medical Sciences Cheeloo College of Medicine Shandong University Jinan Shandong 250012 China; ^4^ Department of Endocrinology and Metabolism The Second Hospital of Shandong University Jinan Shandong 250033 China; ^5^ Department of Medical Psychology and Ethics School of Basic Medical sciences Cheeloo College of Medicine Shandong University Jinan Shandong 250012 China

**Keywords:** depression, dorsal raphe nucleus, exercise, medial prefrontal cortex, synaptic plasticity

## Abstract

Exercise is considered as playing a pivotal role in the modulation of emotional responses. However, a precise circuit that mediates the effects of exercise on depression have yet to be elucidated. Here, a molecularly defined tri‐synaptic pathway circuit is identified that correlates motor inputs with antidepressant effects. With this pathway, initial inputs from neurons within the dorsal root ganglia (DRG) project to excitatory neurons in the gracile nucleus (GR), which in turn connect with 5‐HTergic neurons in the dorsal raphe nucleus (DRN), eventually coursing to excitatory pyramidal neurons within the medial prefrontal cortex (mPFC). Exercise activates this pathway, with the result that depressive‐ and anxiety‐like behaviors in mice are significantly reduced. In addition, it is found that exercise may exert antidepressant effects through regulating synaptic plasticity within this tri‐synaptic pathway. These findings reveal a hindbrain‐to‐forebrain neuronal circuit that specifically modulates depression and provides a potential mechanism for the antidepressant effects of exercise.

## Graphical abstract

During exercise, the GR directly stimulates the DRN, which then regulates the mPFC of in limbic system regions by releasing serotonin (5‐HT) to alleviate stress‐induced depressive states. Sustained aerobic exercise promotes synaptic plasticity within the nuclei of this pathway, thereby prolonging and amplifying the antidepressant effects of physical exercise.

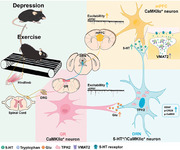

## 1 Introduction

Major depressive disorder (MDD) is a highly heterogeneous syndrome impacting over 300 million individuals worldwide.^[^ ^1^ ^]^ Its etiology is multifactorial, encompassing genetic, psychological, environmental, and social dimensions, and is frequently accompanied with comorbidities.^[^ ^2^, ^3^, ^4^, ^5^ ^]^ As a pervasive mental health issue, MDD substantially compromises the quality of life in these individuals and is projected to ascend as the primary global disease burden by 2030.^[^ ^6^, ^7^ ^]^ However, the underlying etiology and mechanisms of depression remain largely elusive, and therapeutic options are markedly limited. Consequently, identifying the neural basis of depression along with the development of preventative and more efficacious treatment strategies represent topics of paramount clinical importance for MDD.

Physical exercise is universally acknowledged for its capacity to impart significant psychological and physical health advantages.^[^ ^8^, ^9^, ^10^, ^11^ ^]^ Movement is generated by corticospinal tracts emanating from the motor cortex and projecting to the corresponding anterior horns of the spinal cord or to the motor nuclei of the brainstem, thereby producing corresponding muscular movements.^[^ ^12^, ^13^, ^14^ ^]^ With movement, proprioceptive information from the lower limbs is transmitted from the dorsal root ganglia (DRG) to the fasciculus gracilis by pseudounipolar neurons. This information is then transmitted to the gracile nucleus (GR), and subsequently along the medial lemniscus tract from the medulla oblongata to the brain, which then integrates inputs from other brain nuclei to produce movement.^[^ ^15^, ^16^, ^17^, ^18^ ^]^ Findings from previous studies have documented that exercise contributes to the mitigation of depressive symptoms via an array of biological processes, including genetic modulation, anti‐inflammation and ‐oxidation, enhancement of primary neurotransmitter synthesis and release, promotion of neuroplasticity and improvement of structural and functional anomalies in crucial brain regions.^[^ ^9^, ^19^, ^20^, ^21^, ^22^, ^23^ ^]^ While the antidepressant effects of physical exercise are well‐established, the precise mechanisms underlying these benefits remain to be elucidated. Therefore, unveiling the structure and function of neuronal circuits implicated in exercise‐induced mood modulation is crucial for a comprehensive understanding of the bases for the ability of physical exercise to influence depressive symptoms.

Serotonin (5‐HT) plays a pivotal role in modulating a variety of physiological and behavioral states, including learning and memory, sensory perception, as well as motor and emotional responses.^[^ ^24^, ^25^, ^26^, ^27^, ^28^, ^29^ ^]^ Selective serotonin reuptake inhibitors (SSRIs) are extensively employed in treating MDD.^[^ ^30^, ^31^ ^]^ The dorsal raphe nucleus (DRN), serving as the central nervous system's primary serotonin source, extensively innervates midbrain and forebrain regions integral to reward processing, emotional responses and sensory‐motor integration.^[^ ^32^, ^33^, ^34^, ^35^ ^]^ These regions mainly encompass the ventral tegmental area (VTA), thalamus, hypothalamus, hippocampus, amygdala, and medial prefrontal cortex (mPFC).^[^ ^36^, ^37^, ^38^, ^39^ ^]^ Such projection pathways are responsive to adverse stimuli, such as stress, and thus elicit antidepressant and anxiolytic responses.^[^ ^37^, ^40^ ^]^ Nonetheless, whether the DRN plays a role in mediating the antidepressant effects of exercise, and the mechanisms by which exercise‐induced limbic nerve impulses are conveyed from the DRN, remain largely unexplored.

Here, we investigated the molecular and circuitous projections of the GR‐DRN‐mPFC pathway. This ascending hindbrain‐forebrain tri‐synaptic pathway, integrates motor inputs associated with emotional responses. Our findings demonstrate a pivotal role for the GR‐DRN‐mPFC pathway in modulating the antidepressant‐like effects of physical exercise. In specific, we found that the GR, acting as an intermediary in movement's upstream pathway, directly stimulates the DRN. This activation then regulates the mPFC of limbic system regions via serotonin (5‐HT) release to alleviate stress‐induced depressive states. The elucidation of this pathway provides novel insights into the neural mechanisms underlying the efficacy of exercise and its benefits as an antidepressant therapy.

## 2 Results

### 2.1 Physical Exercise Decreases Depressive‐Like Behaviors in Chronic Stress Mouse Models

To assess the antidepressant potential of exercise, we employed an optimal aerobic training velocity for treadmill exercise in mice as achieved with use of an incremental load test (ILT) (Figure S1A–C, Supporting Information). Based on ILT results, the treadmill speed for mice was set at 10 m min^−1^. Two different chronic stress mouse models, chronic restraint stress (CRS) and chronic social defeat stress (CSDS), were then used to assess the antidepressant efficacy of exercise. Stress‐induced behavioral changes in these mice were assessed using the sucrose preference test (SPT) and forced swimming test (FST), while anxiety‐like behaviors were assessed via the open field test (OFT) and elevated plus maze (EPM).

**Figure 1:**
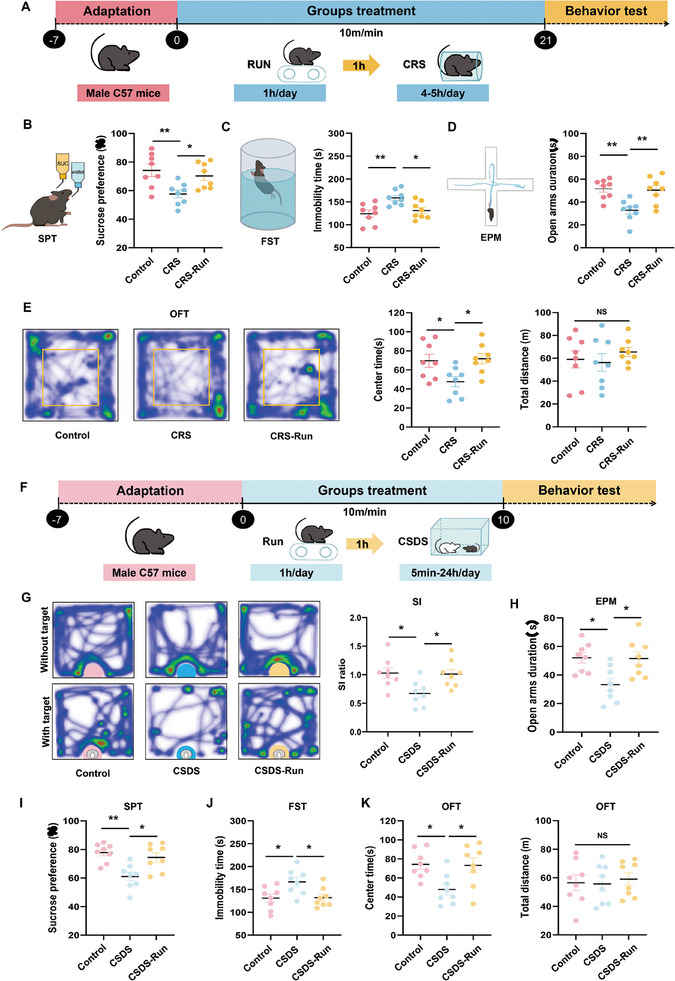
Exercise decreases depressive‐like behaviors induced by chronic stress. A) Schematic of the experimental design for the CRS model. B–E) Depressive‐ and anxiety‐like behaviors in the different experimental groups of the CRS model (*n* = 8 animals per group). SPT (B), FST (C), EPM (D), and OFT (E). F) Schematic of the experimental design for the CSDS model. G) Social interactions in the different experimental groups of the CSDS model. H–L) Depressive‐, stress‐induced‐, and anxiety‐like behaviors in the different experimental groups of the CSDS model (*n* = 8 animals per group). EPM (H), SPT (I), FST (J), and OFT (K). For all statistical tests: One‐way analysis of variance (ANOVA) with Bonferroni post‐hoc test. *, *P* < 0.05; **, *P* < 0.01; NS, no significant difference. Data are presented as Means ± SEMs. Dots represent individual mice.

For the CRS model, mice underwent a daily 1 h treadmill exercise regime for 21 days. The running speed of 10 m min^−1^ to achieve the intended aerobic training intensity (**Figure** **1**A). Results from the behavioral tests as conducted after the three weeks of exercise revealed that depression‐ and anxiety‐like behaviors were significantly alleviated. In specific, these exercised CRS mice showed increased preferences for sugar water in the SPT, decreased immobility times in the FST, significant increases in times and entries into the open arms of the EPM and time in the central area of the OFT (Figure 1B–E).

For the chronic social defeat stress (CSDS) model (described below) and the non‐stressed controls, mice were subjected to adaptive treadmill training prior to induction of the CSDS model. During the modeling phase, a 1 h regime of treadmill exercise was administered prior to the social defeat interaction with CD‐1 mice (Figure 1F). Results of the behavioral assessments, as conducted 10 days later, demonstrated that exercise also ameliorated the depression‐ and anxiety‐like behaviors of these CSDS mice (Figure 1G–K).

### 2.2 Exercise Activates a Direct Projection from the GR to the DRN

**Figure 2:**
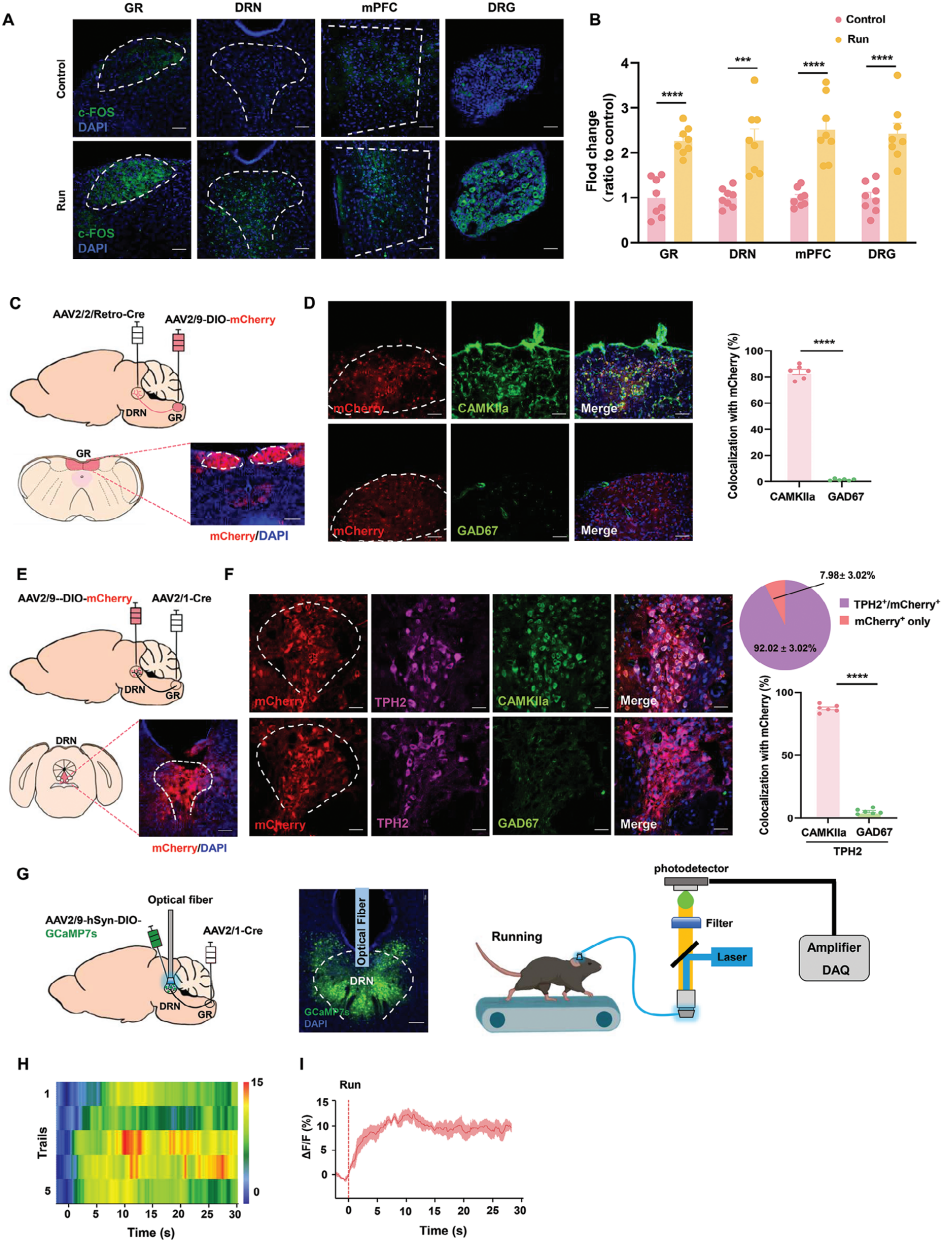
Exercise‐induced activation of GR CaMKII‐α^+^ neurons directly triggers DRN 5‐HT^+^∩CaMKII‐α^+^ neurons. A) Typical image of Fos expression in DRG, GR, DRN, and mPFC. Scale bar: 50 µm. B) Quantification of c‐Fos+ cell numbers in control and runing mice (*n* = 8 mice per group). C) Schematic of virus injection and typical images of mCherry expression in GR. Scale bar, 100 µm. (*n* = 6 mice per group). D) Typical image and quantification of DRN‐projecting GR neurons co‐expressed with CaMKII/GAD2 and quantification percent. Scale bar: 20 µm. (*n* = 6 mice per group). E) Schematic of virus injection and typical images of mCherry expression in DRN. Scale bar: 100 µm. F) Typical image and quantification of DRN postsynaptic neurons co‐expressed with CaMKII∩TPH2 or GAD2∩TPH2 and quantification percent. Scale bar: 20 µm. G) Schematic for monitoring calcium activities of GR‐projecting DRN. Scale bar: 100 µm. H,I) Heatmap H) and quantifications (I) of calcium signal changes of GR‐projecting DRN, as aligned to the onset (time = 0 s) of running (*n* = 5 bouts from 5 mice). Two‐tailed unpaired Student's *t*‐test for (B), (D), and (F). ***p < 0.001; ****p < 0.0001; NS, not significant. Data are presented as Means ± SEMs.

To identify specific brain regions implicated in the antidepressant effects of exercise, we employed c‐Fos as a means to evaluate neuronal activity in critical regions involved in the ascending transmission of exercise and emotional responses. The DRN exhibited significantly heightened c‐Fos signals in the exercise group as compared to controls. Pronounced markers were also detected in other nuclei associated with exercise, such as the dorsal root ganglia (DRG), gracile nucleus (GR), as well as in notable regions associated with stress responses, such as the medial prefrontal cortex (mPFC) (**Figure** **2**A,B), hippocampus, lateral habenular nucleus (LHb) and amygdala (Figure S2A,B, Supporting Information).

As a relay station for proprioception, the GR mediates upstream afferents within the sensory pathway.^[^ ^41^ ^]^ Our initial investigations focused on determining whether DRN received direct projections from the GR in these mice. We observed an extensive amount of fiber projections in the DRN following the injection of AAV2/9‐CAG‐EGFP‐WPRE into the GR region, while the expression of mCherry in the GR was noted upon injecting AAV2/Retro‐CAG‐mCherry‐WPRE into the DRN (Figure S2C–E, Supporting Information). We also verified the projection of DRG‐GR by tracing red retro‐beads (Figure S2F, Supporting Information).

The GR possesses a significant population of excitatory neurons.^[^ ^42^, ^43^ ^]^ To establish the types of GR neurons projecting to the DRN, GR neurons were targeted within the GR‐DRN pathway by injecting AAV2/Retro‐hSyn‐cre into the DRN and AAV2/9‐DIO‐mCherry into the GR (Figure 2C). Results from these injections revealed that 83.8 ± 4.36% of these mCherry‐labeled GR neurons were also co‐labelled with CaMKII‐α (Figure 2D). It has been reported that along with the presence of other neuronal types found in the DRN, such as GABAergic, dopaminergic, and glutamatergic neurons, the DRN harbors the majority of 5‐HT neurons projecting to the forebrain.^[^ ^44^, ^45^ ^]^ To further evaluate the projection relationship and cellular properties of the GR‐DRN pathway, we administered the monosynaptic anterograde transport virus, AAV2/1‐hSyn‐Cre, to the GR and a cre‐dependent virus encoding the red fluorescent protein mCherry (AAV2/9‐DIO‐mCherry) to the DRN in C57BL/6 mice (Figure 2E). Remarkably, ≈87.25 ± 2.7% of DRN postsynaptic neurons expressing mCherry were immunoreactive to CaMKII‐α and TPH2, indicating that these were excitatory 5‐HTergic neurons (Figure 2F).

Subsequently, we employed fiber photometry to precisely track the neural dynamics of the GR‐DRN pathway. This was achieved by tagging GR‐DRN neurons with AAV2/1‐hSyn‐cre and a cre‐dependent virus encoding the calcium indicator jGCaMP7s (AAV2/9‐DIO‐jGCaMP7s) (Figure 2G). Upon initiation of exercise in these mice, their neuronal activity within the DRN showed a time‐locked increase and maintained plateau of activity (Figure 2H,I; Figure S2G–I, Supporting Information). Accordingly, these findings delineate the GR ^CaMKII‐α^ **^+^** – DRN ^5‐HT^ **^+^** ^∩CaMKII‐α^ **^+^** circuit, as a circuit that is activated with treadmill exercise and represents a critical neural link between motor upregulation and emotional responses.

### 2.3 Exercise Input to DRN Drives mPFC Activation

**Figure 3:**
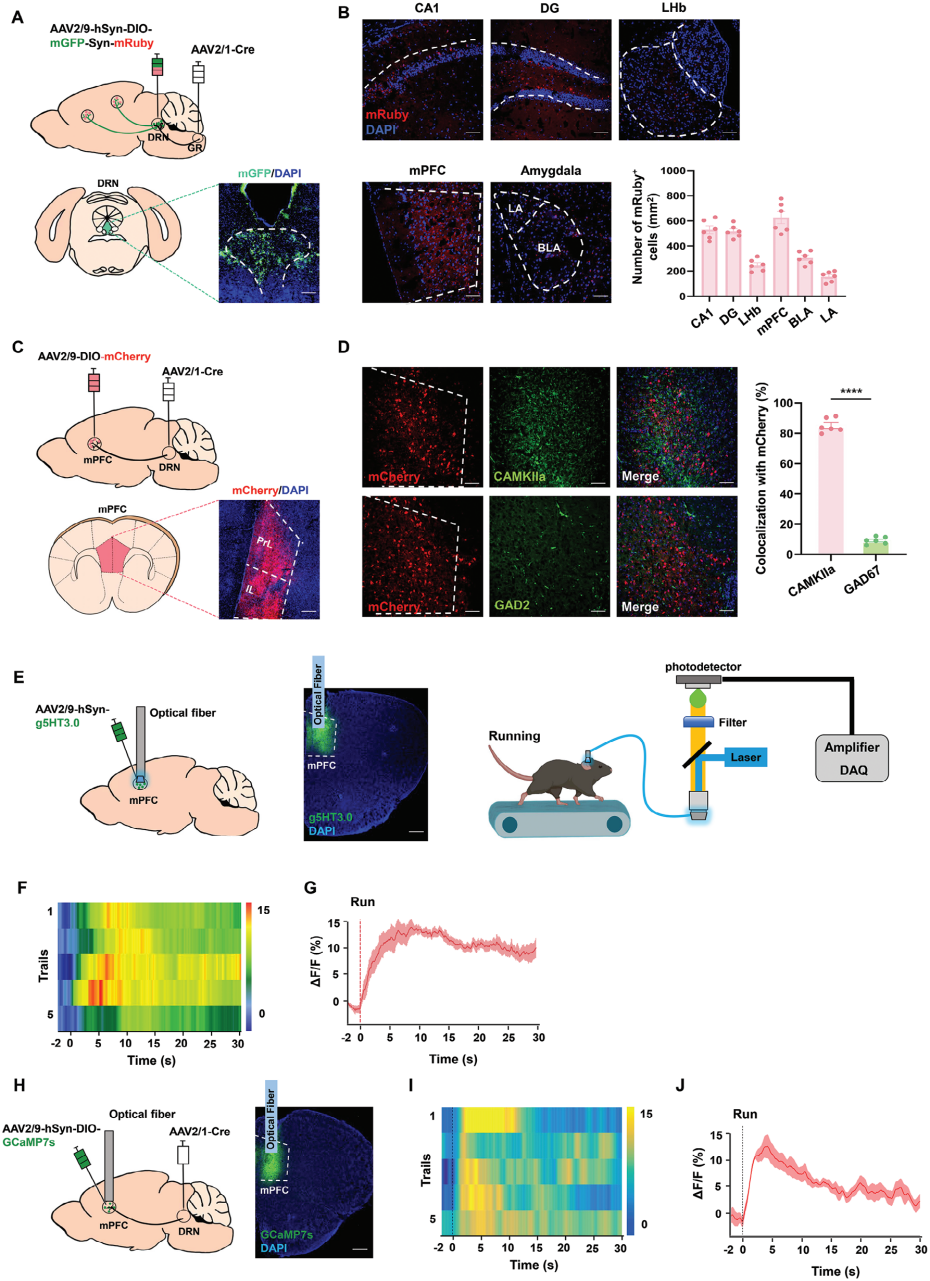
The GR‐DRN pathway directly innervates mPFC CaMKII‐α^+^ neurons. A) Experimental design used for labeling axons and presynaptic terminals of DRN postsynaptic neurons. Representative image of injection site in DRN. Scale bar: 100 µm. B) Synaptic puncta in the mPFC, CA1, DG, LHb, and amygdala from DRN postsynaptic neurons. Scale bar: 50 µm (*n* =  6 brain slices from 3 mice per group). C) Schematic of virus injection and typical images of mCherry expression in the mPFC. Scale bar, 100 µm. D) Typical image and quantification of DRN‐projecting mPFC neurons co‐expressed with CaMKII/GAD2 and quantification percent. Scale bar: 50 µm (*n* = 6 mice per group). E) Schematic depicting recordings of 5‐HT levels in the mPFC and representative image of virus expression. Scale bar: 100 µm. F,G) Heatmap (F) and quantifications (G) of 5‐HT level changes in DRN‐projecting mPFC neurons, as aligned to the onset (time = 0 s) of running (*n* = 5 bouts from 5 mice). H) Schematic for monitoring calcium activities of DRN‐projecting mPFC. Scale bar: 100 µm. I‐J) Heatmap (I) and quantifications (J) of calcium signal changes of DRN‐projecting mPFC, as aligned to the onset (time = 0 s) of running (*n* = 5 bouts from 5 mice). Two‐tailed unpaired Student's *t*‐test for (D). ****p < 0.0001; Data are presented as Means ± SEMs.

To identify the projection targets of DRN postsynaptic neurons, we initially labeled axons and presynaptic terminals by injecting AAV2/1‐hSyn‐Cre into the GR and AAV2/9‐hSyn‐DIO‐mGFP‐T2A‐Synaptophysin‐mRuby into the DRN (**Figure** **3**A). Results of these procedures revealed that neurons in the DRN receiving GR inputs project to various emotion‐related limbic system regions, including the prefrontal cortex, hippocampus, amygdala, and lateral habenula (LHb) (Figure 3B). Notably, channels expressing mCherry were predominantly observed in the mPFC. Moreover, mPFC postsynaptic neurons receiving afferent projections from DRN were infected with AAV2/9‐DIO‐mCherry, and ≈85,1 ± 4.29% of these mPFC mCherry‐expressing neurons were identified as excitatory neurons (Figure 3C,D; Figure S3A, Supporting Information). Such findings indicate that during exercise, 5‐HT neurons within DRN facilitate activation of the mPFC through monosynaptic excitatory connections, supporting an involvement of the molecularly defined GR ^CaMKII‐α+^ – DRN ^5‐HT+∩CaMKII‐α+^‐ mPFC ^CaMKII‐α+^ pathway.

To track dynamic changes of postsynaptic serotonin (5‐HT) release from DRN 5‐HT neurons in the mPFC during exercise, we employed a serotonin‐specific fluorescent probe virus (g5HT3.0). With this probe 5‐HT concentrations correlate with the fluorescent intensity of green fluorescent protein (GFP). After injecting AAV‐hSyn‐g5HT3.0 into the mPFC and employing fiber photometry and tagging GR‐DRN neurons with AAV2/1‐hSyn‐cre and a cre‐dependent virus encoding the calcium indicator jGCaMP7s, we observed an increase in the g5HT3.0 signal and jGCaMP7s signal within the mPFC as associated with treadmill exercise (Figure 3E,H). These findings suggest that activation of DRN 5‐HT neurons during physical activity, result in the release (Figure 3F,G; Figure S3B–D, Supporting Information) and consequent activation of 5‐HT in the mPFC (Figure 3I,J).

### 2.4 Activation of the GR‐DRN‐mPFC Pathway Reduces Depression‐Like Behavior

**Figure 4:**
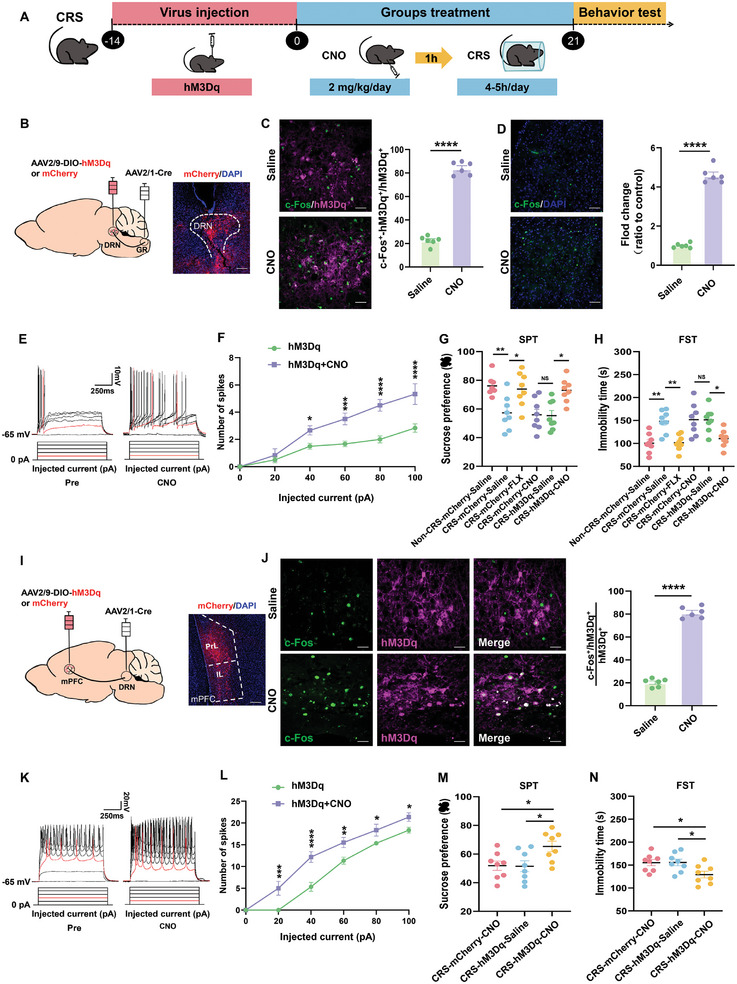
Activation of the GR‐DRN‐mPFC pathway decreases depressive‐like behaviors induced by chronic restraint stress. A) Schematic of the experimental design for the CRS model. B) Schematic of virus injection and typical images of mCherry expression in DRN. Scale bar: 100 µm. C) Representative images and quantification of DRN showing c‐Fos expression in neurons expressing hM3Dq in response to an i.p. injection of saline or CNO. Scale bar: 20 µm (*n* = 6 mice per group). D) Representative images and quantification of mPFC showing c‐Fos expression in response to an i.p. injection of saline or CNO. Scale bar: 50 µm (*n* = 6 mice per group). E) Representative traces of voltage responses in DRN mCherry^+^ neurons to injection currents from 0 to 100 pA with 20 pA steps before and after bath application of CNO (5 µm). Red traces indicate minimal currents needed to induce APs. F) CNO increases spiking of hM3Dq‐expressing neurons (*n* = 6 cells from 3 mice per group). G,H) Depressive‐like and stress‐induced behaviors in the different experimental groups of the CRS model (*n* = 8 animals per group). SPT (G) and FST (H). I) Schematic of virus injection and typical images of mCherry expression in the mPFC. Scale bar: 100 µm. J) Representative images and quantification of the mPFC showing c‐Fos expression in neurons expressing hM3Dq in response to an i.p. injection of saline or CNO. Scale bar: 20 µm. *N *= 6 mice per group. K) Representative traces and analyses of voltage responses in mPFC mCherry^+^ neurons to injection currents from 0 to 100 pA with 20 pA steps before and after bath application of CNO (5 µm). Red traces indicate minimal current needed to induce APs. L) CNO decreases spiking of hM3Dq‐expressing neurons (*n* = 6 cells from 3 mice per group). M,N) Depressive‐like behaviors in the different experimental groups of the CRS model (*n* = 8 animals per group). SPT (M) and FST (N). Two‐tailed unpaired Student's *t*‐test for (C), (D), and (J) and paired Student's *t*‐test for (F) and (L). For (G), (H), (M), and (N): One‐way analysis of variance (ANOVA) with Bonferroni post‐hoc test. *, *P* < 0.05; **, *P* < 0.01; ***p < 0.001; ****p < 0.0001; NS = no significant difference. Data are presented as Means ± SEMs.

To determine the function of the GR‐DRN‐mPFC pathway in modulating depressive behaviors in these chronically stressed mice (**Figure** **4**A), we injected AAV2/1‐hSyn‐Cre into the GR and targeted GR‐projecting DRN with either AAV2/9‐DIO‐hM3Dq‐mCherry or AAV2/9‐DIO‐mCherry (Figure 4B). Following these treatments an intraperitoneal injection clozapine N‐oxide (CNO) was administered to activate these neurons (Figure 4C,D). Electrophysiological assessments indicated a reduction in firing rates of GR‐projecting to DRN after CNO (Figure 4E,F,K,L). As another approach to evaluate this function, during the 3‐week period of CRS GR‐projecting DRN were activated as achieved via daily intravenous CNO injection (2 mg kg^−1^) before stress modeling. Activation of DRN receiving GR projections significantly alleviated depression‐ and anxiety‐like behaviors compared to that in the saline‐injected control group, an alleviation that was comparable to that observed with a daily 21‐day administration of fluoxetine (As a positive control drug for antidepressant treatment, i.p. 10 mg kg^−1^) (Figure 4G,H; Figure S5C,D, Supporting Information). In addition, chemogenetic stimulation of the DRN‐mPFC pathway followed by activation of DRN‐projecting mPFC neurons markedly diminished both depression‐ and anxiety‐like behaviors (Figure 4M,N; Figure S6C,D, Supporting Information). These results align with the behavioral improvements observed in response to activation of the DRN‐projecting GR neurons in mice (Figure S4, Supporting Information).

We extended these investigations to examine the GR‐DRN‐mPFC pathway as mediating anti‐depressive and anti‐anxiety effects within the CSDS model. Throughout the 10‐day period of CSDS, CNO was administered daily to activate either the GR‐DRN pathway (Figure S5E–K, Supporting Information) or DRN‐projecting mPFC neurons (Figure S6E–K, Supporting Information). Notably, stimulation of the GR‐DRN‐mPFC pathway resulted in a significant reduction of depression‐ and anxiety‐like behaviors. Collectively, these findings suggest that activation of the GR‐DRN‐mPFC pathway effectively alleviates depression‐ and anxiety‐like behaviors as induced in these CRS and CSDS mouse models.

### 2.5 Inhibition of the GR‐DRN‐mPFC Pathway Prevents Exercise‐Induced Antidepressant Effects

**Figure 5:**
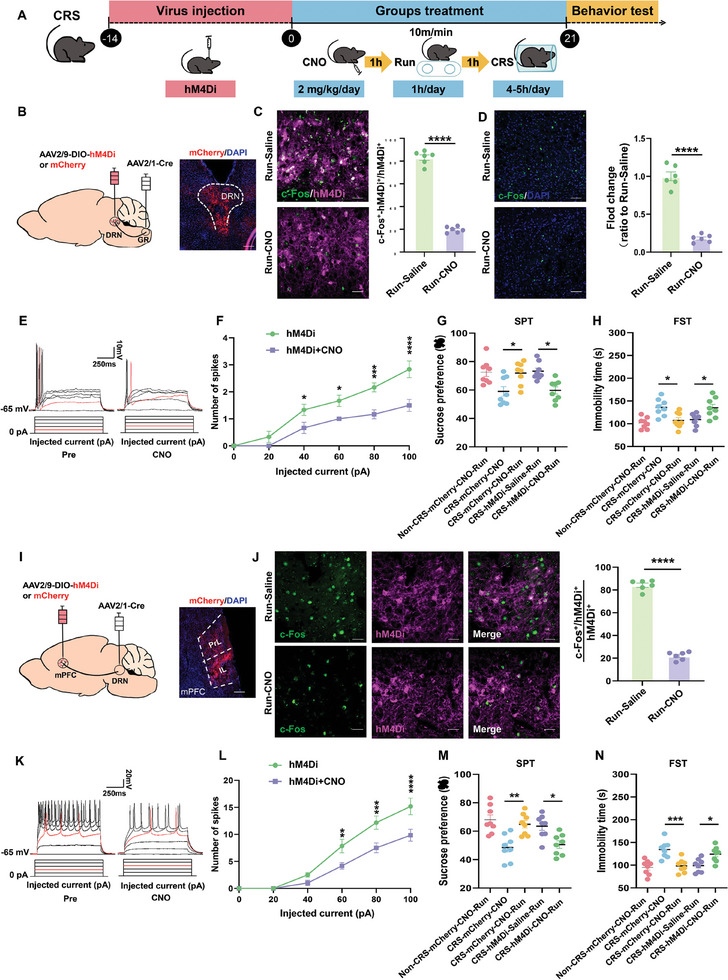
Inhibition of the GR‐DRN‐mPFC pathway prevents exercise‐induced antidepressant effects. A) Schematic of the experimental design for the CRS model. B) Schematic of virus injection and typical images of mCherry expression in DRN. Scale bar: 100 µm. C) Representative images and quantification of DRN showing c‐Fos expression in neurons expressing hM4Di in response to an i.p. injection of saline or CNO. Scale bar: 20 µm (*n* = 6 mice per group). D) Representative images and quantification of mPFC showing c‐Fos expression in response to an i.p. injection of saline or CNO. Scale bar: 50 µm (*n* = 6 mice per group). E) Representative traces of voltage responses in DRN mCherry^+^ neurons to injection currents from 0 to 100 pA with 20‐pA steps before and after bath application of CNO (5 µm). Red traces indicate minimal currents needed to induce APs. F) CNO increases spiking of hM4Di‐expressing neurons (*n* = 6 cells from 3 mice per group). G,H) Depressive‐like and stress‐induced behaviors in the different experimental groups of the CRS model (*n* = 8 animals per group). SPT (G) and FST (H). I) Schematic of virus injection and typical images of mCherry expression in the mPFC. Scale bar: 100 µm. J) Representative images and quantification of the mPFC showing c‐Fos expression in neurons expressing hM4Di in response to an i.p. injection of saline or CNO. Scale bar: 20 µm (*n* = 6 mice/group). K) Representative traces and analyses of voltage responses in mPFC mCherry^+^ neurons to injection currents from 0 to 100 pA with 20 pA steps before and after bath application of CNO (5 µm). Red traces indicate minimal currents needed to induce APs. L) CNO decreases spiking of hM4Di‐expressing neurons (*n* = 6 cells from 3 mice per group). M,N) Depressive‐like behaviors in the different experimental groups of the CRS model (*n* = 8 animals/group). SPT (M) and FST (N). Two‐tailed unpaired Student's *t*‐test for (C), (D), and (J) and paired Student's *t*‐test for (F) and (L). For (G), (H), (M), and (N): One‐way analysis of variance (ANOVA) with Bonferroni post‐hoc test. *, *P* < 0.05; **, *P* < 0.01; ***p < 0.001; ****p < 0.0001; NS = no significant difference. Data are presented as Means ± SEMs.

To further assess involvement of the GR‐DRN‐mPFC pathway as related to the antidepressant effects of exercise, we employed chemical genetics to inhibition this pathway. In specific, AAV2/1‐Cre was administered into the GR and AAV2/9‐DIO‐hM4Di‐mCherry was used to target DRN neurons. Mice received CNO injections prior to daily stress modeling, followed by treadmill exercise 30 min later (**Figure** **5**A). Both c‐FOS expression and electrophysiological analyses demonstrated that this CNO administration effectively inhibited the activity of neurons expressing hM4Di as well as the activity of activated neurons during exercise (Figure 5B–F). Furthermore, inhibiting DRN receiving projections from GR nullified the antidepressant effects of exercise, as evidenced by a reduction in sucrose water preference in the SPT, increases in immobility times in the FST, and decreases in durations within the central area of the OFT and frequencies/times of open arm entries in the EPM (Figure 5G,H; Figure S8, Supporting Information).

We next utilized chemical genetics to suppress the DRN‐mPFC pathway to investigate its role in exercise‐induced antidepressant effects. AAV2/1‐Cre was administered into the DRN and AAV2/9‐DIO‐hM4Di‐mCherry into mPFC regions (Figure 5I–L). This targeted inhibition of mPFC neurons effectively nullified the antidepressant benefits of exercise in both CRS and CDSD mice (Figure 5M,N; Figure S9, Supporting Information).

Finally, we investigated the necessity of GR neurons projecting to the DRN for alleviating depressive symptoms as achieved by activating these neurons during exercise. To selectively inhibit GR neurons projecting to DRN, rAAV2/2‐Retro‐cre was introduced into DRN and while AAV2/9‐DIO‐hM4Di‐mCherry was injected into the GR region (Figure S7A–D, Supporting Information). Inhibition of GR‐to‐DRN projections during exercise also abrogated the antidepressant effects of physical activity (Figure S7E–O, Supporting Information). These findings provide robust evidence indicating that activation of the GR‐DRN‐mPFC pathway is essential for the mitigation of depressive behaviors resulting from CRS or CSDS in mice.

### 2.6 Long‐Term Exercise Improves Depressive Symptoms by Regulating Synaptic Plasticity

**Figure 6:**
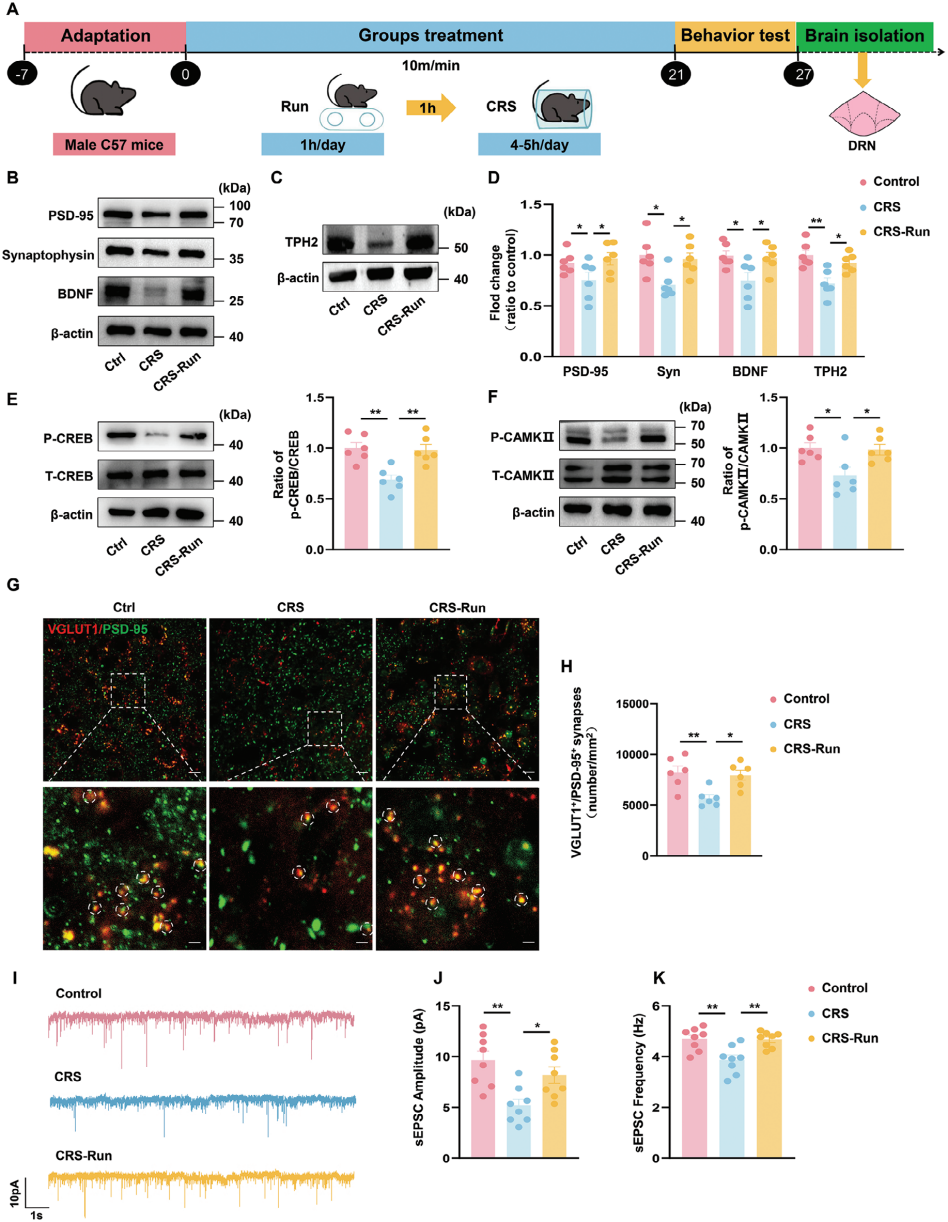
Exercise promotes synaptic plasticity of neurons within DRN. A) Schematic of the experimental design for the CRS model. B,C) Representative western blots showing that exercise increased expression levels of synaptic structural proteins (B) and TPH2 (C) in DRN of mice exposed to CRS. D) Quantification of protein expression levels of PSD95, Synaptophysin, BDNF and TPH2 within the DRN region. E,F) Representative western blots and quantification of p‐CREB/CREB (E) and P‐CAMKII/CAMKII (F) within the DRN region. G) Immunofluorescent staining showing VGLUT1^+^ (red) and PSD95^+^ (green) co‐localization in DRN neurons. Top: scale bar: 10 µm. Bottom: scale bar: 5 µm (*n *= 6 brain slices from 3 mice for each group). H) The quantification of the synaptic number in DRN with each group. I) Representative traces of sEPSCs in DRN neurons with each group. J,K) Cumulative fraction plots of sEPSCs amplitude (J) and frequencies(K) (*n* = 8 cells from 3 mice/group). For (B‐F): *n* = 6 animals/group. One‐way analysis of variance (ANOVA) with Bonferroni post‐hoc test. *, *P* < 0.05; **, *P* < 0.01; NS, no significant difference. Data are presented as Means ± SEMs.

We have demonstrated that the physical activity involved with treadmill exercise in mice ameliorated depression‐like behavior through stimulation of the GR‐DRN‐mPFC pathway. Whether this sustained exercise may further augment neuroplasticity, concomitant with a plethora of cellular and molecular changes was the issue to be addressed in our next series of experiments. Results from both clinical and animal studies have indicated that an upregulation of brain derived neurotrophic factor (BDNF) during exercise plays a pivotal role in modulating synaptic plasticity.^[^ ^46^ ^]^ Here, we found that exercise enhanced BDNF expression, phosphorylation of cAMP‐response element binding protein (CREB) and calmodulin dependent protein kinsae II (CAMKII), along with an upregulation of the synaptic structural proteins, post synaptic density 95 (PSD95) and synaptophysin (SYN), in CRS and CSDS mice (**Figure** **6**B–F; Figure S10B–F, Supporting Information). This indicates that long‐term exercise fosters synaptic plasticity in the DRN of the mouse brain. Supporting these findings are results from western blots, where we also observed a significant increment in the number of Vglut1^+^/PSD95^+^ synapses within the DRN in exercised versus non‐exercised control CRS and CSDS mice (Figure 6G,H; Figures S10G,H and S12A,B, Supporting Information). To further explore whether the increase of structural synaptic were accompanied by functional changes in DRN neurons, we conducted whole‐cell patch‐clamp recordings, with the results that exercise increased the frequency and amplitude of spontaneous excitatory postsynaptic current (sEPSC) (Figure 6I–K). In addition, the findings that exercise increased tryptophan hydroxylase 2 (TPH2) expression within the DRN, suggests an enhancement in 5‐HT synthesis (Figure 6C; Figure S10C, Supporting Information). Based on these results, it appears that prolonged exercise culminates in improved synaptic plasticity within the DRN, characterized by an augmented number of functional glutamatergic synapses receiving projections from the GR, along with an amplified synthesis of 5‐HT.

**Figure 7:**
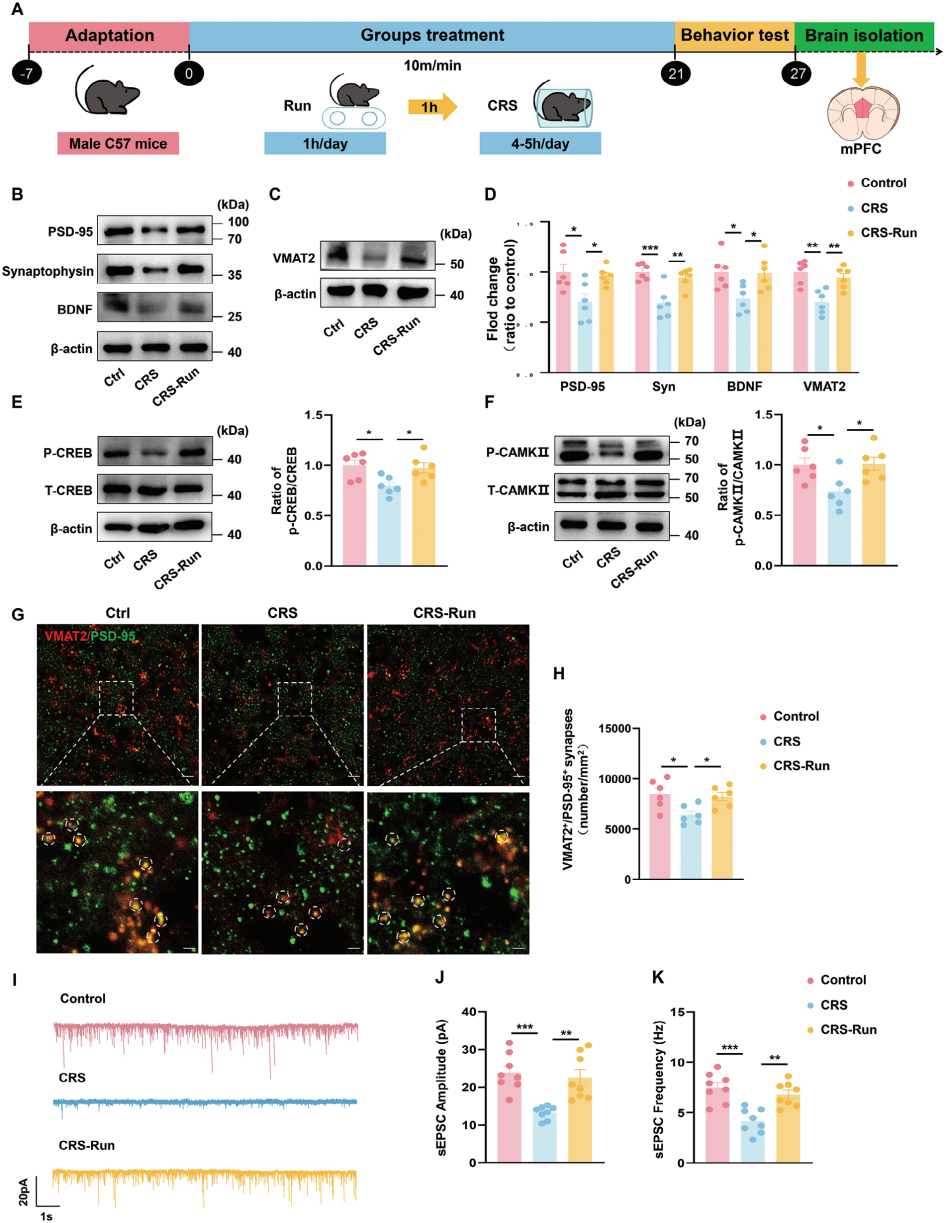
Exercise promotes synaptic plasticity of neurons within the mPFC. A) Schematic of the experimental design for the CRS model. B,C) Representative western blots showing that exercise increased expression levels of synaptic structural proteins (B) and TPH2 (C) in the mPFC of mice exposed to CRS. D) Quantification of protein expression levels of PSD95, SYN, BDNF and TPH2 within the mPFC region. E,F) Representative western blots and quantification of p‐CREB/CREB (E) and P‐CAMKII/CAMKII (F) within the mPFC region. G) Immunofluorescent staining showing VMAT2^+^ (red) and PSD95^+^ (green) co‐localization in DRN neurons. Top: scale bar: 10 µm. Bottom: scale bar: 5 µm (*n* = 6 brain slices from 3 mice for each group). H) The quantification of the synaptic number in mPFC with each group. I) Representative traces of sEPSCs in mPFC neurons with each group. J,K) Cumulative fraction plots of sEPSCs amplitude (J) and frequencies(K) (*n *= 8 cells from 3 mice per group). For (B‐F): *n* = 6 animals per group. One‐way analysis of variance (ANOVA) with Bonferroni post‐hoc test. *, *P* < 0.05; **, *P* < 0.01; NS, no significant difference. Data are presented as Means ± SEMs.

We also observed a similar increase in the expression of proteins associated with synaptic plasticity within the mPFC, suggesting that sustained exercise also enhances synaptic plasticity at this site (**Figure** **7**B–F; Figure S11B–F, Supporting Information). When evaluating expression levels of the vesicle monoamine transporter protein 2 (VMAT2) within the mPFC, we found these levels to be increased in exercised versus non‐exercised CRS and CSDS mice (Figure 7C; Figure S11C, Supporting Information). A significant proliferation in VMAT2^+^/PSD95^+^ synapses were observed in the former group (Figure 7G,H; Figure S11G,H, Supporting Information), and the frequency and amplitude of sEPSC were increased by exercise (Figure 7I–K). These findings reveal a positive link between physical activity and the augmentation of monoamine transmitter release, along with synapse development in the mPFC. Collectively, these results suggest that physical exercise may exert long‐term antidepressant effects through the modulation of synaptic plasticity within the GR‐DRN‐mPFC pathway.

## 3 Discussion

In the current era of contemporary life, the pressures resulting from our environment and interpersonal relationships frequently lead to a spectrum of emotional disorders.^[^ ^47^ ^]^ One suggested coping mechanism to achieve a more balanced lifestyle involves regular exercise, which can mitigate some of these psychological disorders.^[^ ^8^, ^9^, ^10^ ^]^ Although the health benefits of exercise are widely acknowledged, the intricacies regarding interactions among ascending sensory pathways with various brain nuclei, and the regulatory dynamics among these circuits, as related to exercise and depression, remain poorly understood. Here, we delineate a pathway wherein an ascending sensory conduction pathway facilitates the modulation of depression. Specifically, DRG neurons of the spinal cord capture afferent impulses emanating from primary neurons associated with proprioceptors of the limbs and trunk during physical exercise and relay this information to the GR within the medulla oblongata.^[^ ^48^ ^]^ Glutaminergic neurons within the GR then transmit these motor signals to 5‐HT neurons in the DRN, which project to CaMKIIα**^+^**‐pyramidal neurons in the mPFC. This cascade of neural transmission information connects movement and emotion to result in a significant amelioration of depression phenotypes as induced by chronic stress.

As a pivotal relay station for the transmission of peripheral proprioceptive and fine tactile sensations to the brain, the GR is capable of receiving impulses from muscles, tendons, joints and cutaneous sensory receptors originating in the lower limbs and lower trunk.^[^ ^48^ ^]^ With the transit of sensory signals from the periphery through the DRG of the spinal cord during physical exercise, primary afferent neurons establish synaptic connections with secondary neurons in the GR located in the dorsal medulla oblongata.^[^ ^17^, ^41^, ^49^ ^]^ With this pathway it is possible to collect and convey of this information into the brain. Our c‐Fos expression data suggest that there is an elevation in neuronal activity within the DRG and GR following exercise. Concurrently, neuronal activity is enhanced in limbic system nuclei implicated in emotion processing, such as DRN, mPFC, hippocampus and the amygdaloid nucleus. It is noteworthy that the DRN, located in the midbrain and adjacent to GR, are the predominant 5‐HT neuronal nuclei in the central nervous system, nuclei which are considered to be intimately associated with psychological disorders including anhedonia, anxiety and depression.^[^ ^24^, ^33^, ^50^ ^]^ DRN are known to receive monosynaptic projections from diverse cell types of nuclei across the brain, highlighting the intricacy and heterogeneity associated with this site.^[^ ^38^, ^51^ ^]^ While it has been demonstrated that DRN receive monosynaptic projections from nuclei related to sensory, motor and behavioral states within the medulla, direct functional pathways linking the DRN and GR remain uncharacterized.^[^ ^36^ ^]^ To assess the existence of crosstalk between ascending proprioceptive signaling and emotional responses as related to physical exercise, we utilized a combination of retrograde and anterograde viral cross‐synaptic labeling techniques. With these procedures it is possible to demonstrate a singular synaptic connection from the GR, an intracerebral relay station facilitating ascending transmission of sensory data, to the DRN, a critical node in mood regulation. As a result, we have identified that a specific population of CaMKIIα**^+^** glutamatergic neurons within the mouse GR capable of directly targeting 5‐HT neurons within the DRN.

A substantial amount of evidence exists indicating that increases in DRN activity are associated with a reduction in depression‐like behaviors.^[^ ^37^, ^50^, ^52^ ^]^ As based on this evidence, we posited that stimulating the GR‐DRN pathway could alleviate such behavioral disorders. To test this hypothesis, we employed chemogenetic techniques to activate GR neurons projecting to the DRN or DRN postsynaptic neurons in two separate mouse models of depression. With this approach we demonstrated that sustained activation of GR neurons projecting to the DRN or DRN‐postsynaptic neurons decreased depressive‐ and anxiety‐like behaviors as induced by CRS or CSDS.

Based on clinical and experimental findings, it has been established that serotonergic circuits originating from DRN are instrumental in modulating depression and anxiety. Results from our viral tracing studies revealed that DRN receiving GR inputs, in turn project to regions such as the mPFC, hippocampus, amygdala and lateral habenula, which are implicated in the regulation of a broad range of behaviors related to mood and cognition, including despair‐like behaviors, anxiety‐like behaviors and learning and memory behaviors.^[^ ^53^, ^54^, ^55^ ^]^ This suggests that the manipulation of the GR‐DRN pathway can produce effects on not only depressive‐like behaviors but also other mood and cognition related behaviors. The mPFC acts as the brain's executive center for the processing of emotional and cognitive information.^[^ ^56^, ^57^, ^58^, ^59^ ^]^ There is experimental evidence demonstrating that there are prevalent postsynaptic neurons within the mPFC are directly connected with terminals of DRN 5‐HT neurons.^[^ ^57^, ^60^ ^]^ Here, we found that chemogenetic activation of the DRN‐mPFC pathway proved efficacious in ameliorating depression‐ and anxiety‐like behaviors within both the CRS and CSDS mouse models of depression.

Simultaneously, we observed that activity of DRN, which receive projections from the GR during exercise, was significantly increased along with an increase in 5‐HT content within the mPFC, as demonstrated using by fiber‐optic techniques. It is well‐documented that 5‐HT activation in DRN triggers the release of a substantial amount of 5‐HT, which then disseminates along axons into the mPFC. We propose that activation of the GR‐DRN‐mPFC pathway during exercise may underly the antidepressant effects of physical activity. Interestingly, the results of fibre optic recording indicated that food reward can also activate neuronal activity in the DRN and increase 5‐HT release in the mPFC. Given that the DRN can be triggered by multiple stimuli and that it plays a role in diverse functions within various circuits, we believe that the activation of the DRN and the change of 5‐HT in the mPFC may also involve the reward effect of exercise training. To test this hypothesis, we administered CNO to our mouse models of depression to chemogenetically inhibit the GR‐DRN‐mPFC pathway and monitored their behavior post‐exercise. With this protocol, we found that suppressing the GR‐DRN‐mPFC pathway diminished the beneficial effects of exercise on the depression‐like behaviors in these mice.

It has been reported that activation of specific neuronal circuits during exercise could rapidly and transiently modulate moods.^[^ ^53^, ^61^, ^62^ ^]^ These findings led us to speculate on, and investigate the impact of, prolonged physical activity on depression in mice. With exercise, synaptic plasticity was enhanced in both DRN and mPFC regions post exercise, highlighted by a marked increase in the number of excitatory functional synapses within the DRN and 5‐HTergic synapses within the mPFC. Significant increases in expression levels of proteins pertinent to synaptic structure and function were also observed. It is proposed that the observed changes in synaptic plasticity are secondary effects of neurons within the GR‐DRN‐mPFC pathway being activated by prolonged exercise. Activation of the GR‐DRN pathway is accompanied by an increase in the transmission of the excitatory neurotransmitter glutamate, which binds to postsynaptic receptors on DRN neurons, thereby increasing the excitability of DRN 5‐HT neurons. Following the activation of the DRN, the release of 5‐HT is increased, which then activates the neurons in the mPFC via binding to 5‐HT receptors.^[^ ^63^ ^]^ The receptor activation may be followed by an increase in intracellular CAMKII expression, which regulates the downstream expression of CREB, BDNF and related synaptic proteins in response to enhanced synaptic plasticity in this region of the pathway after prolonged exercise.^[^ ^64^, ^65^ ^]^ Moreover, our findings that the substantially increased expression levels of TPH2 within the DRN and the increased levels of VMAT2 within the mPFC, suggested that the sustained boosts in DRN 5‐HT‐synthesis and mPFC 5‐HT‐release resulting from exercise amplify these beneficial effects upon depression.

Depression is a multifaceted disorder that involves the potential interplay of gene‐environment and gene‐sex factors.^[^ ^66^, ^67^ ^]^ Simultaneously, gender differences exist in the impact of exercise on energy metabolism, hormone levels, and brain structure and function.^[^ ^67^, ^68^, ^69^ ^]^ It should be noted that this study was exclusively conducted on male mice, necessitating further investigation into the effects of exercise on different genders in future studies. Within this study, we observed that exercise‐mediated GR‐DRN‐mPFC pathway regulated depressive mood and facilitate synaptic plasticity within this pathway's brain region. In view of the multi‐brain regions response during exercise and the diversity of DRN functions, the detailed mechanism through which this pathway enhances plasticity in the development of depression, as well as the function of other brain region projections downstream of the GR‐DRN, requires further exploration.

In conclusion, our current results enabled us to delineate a tri‐synaptic pathway involving the GR‐DRN‐mPFC pathway, which contributes to the antidepressant effects of exercise. These findings reveal that even passive treadmill exercise can be effective in improving mood disorders, at least partially through a direct relay of peripheral ascending sensory information. Moreover, results from these experiments indicate that sustained aerobic exercise promotes synaptic plasticity within the nuclei of this pathway, thereby prolonging and amplifying the antidepressant efficacy of physical exercise. In this way, long‐term aerobic exercise may offer considerable benefits for stress relief and mood regulation. Taken together, the findings of this present study substantially broaden our understanding of the antidepressant mechanisms resulting from exercise, as well as underscore the importance of promoting mental health awareness and the adoption of regular exercise routines.

## 4 Experimental Section

### Animals

All experiments were approved by the Shandong University Institutional Animal Care and Use Committee (ECSBMSSDU‐2022‐2‐65) and were guided by the Association for Assessment and Accreditation of Laboratory Animal Care International. Unless otherwise indicated, adult (6–8 weeks old) male C57BL/6 mice were randomly divided into control and experimental groups and housed in a 22 °C, 12 h light‐dark cycle with food and water supplied ad libitum.

### Chronic Stress Models—Chronic Restraint Stress (CRS)

Mice were restrained daily for 4–5 h for 21 days as previously described.^[^ ^70^, ^71^ ^]^ Briefly, mice were individually placed within a ventilated restraint tube, ensuring that they could not move forward or backward nor reverse within the tube. After 4–5 h, mice were freed from the restraint tube and returned to their home cage.

### Chronic Stress Models—Chronic Social Defeat Stress (CSDS)

The CSDS paradigm was consistent with that described in previous studies with minor modifications.^[^ ^72^ ^]^ Initially, the CD‐1 aggressor mice were screened for 3 consecutive days and then were housed individually in social defeat cages. The C57BL/6 mice were placed in the home cage and adjacent to the CD‐1 mice and subjected to physical interactions with the unfamiliar, aggressive CD‐1 mouse for 10 min. After 10 min of physical contact, the mice were separated by a perforated plexiglass partition, but maintained in sensory contact for 24 h. After 10 days of this CSDS protocol, the animals were housed separately. Control animals were kept in pairs on either side of the social defeat cage without being physically exposed to the CD‐1 mice. Behavioral procedures such as the social interaction test were then used to evaluate the depression‐like behavior of the mice at 24 h after their last social defeat stress.

### Exercise Paradigm

After the mice acclimated to the treadmill apparatus, an incremental load tests were performed as described previously.^[^ ^73^, ^74^ ^]^ Initially using a speed of 6 m min^−1^ and an acceleration of 0, the speed was subsequently increased by 3 m min^−1^ every 3 min until the mice were exhausted. Mice contacting the end of the treadmill 5 times in 1 min were defined as exhausted. The intensity of the aerobic training program was determined by the exhaustion velocity (EV; m min^−1^) with aerobic training velocity = average velocity of exhaustion in the force exhaustion model * 60%.

For the CRS model, the mice were run for 1 h at 10 m min^−1^, Restraints were then applied 1 h after the run. For the CSDS model, the mice were run for 1 h at 10 m min^−1^, before physical contact with the CD‐1 mice.

### Stereotaxic Surgery and Fiber Implantation

Procedures involved with the stereotaxic surgery were performed as described in a previous study.^[^ ^75^ ^]^ Mice were anesthetized (sodium pentobarbital at 35 mg k^−1^g, i.p.) and placed in a stereotaxic apparatus (Stoelting, USA). Viruses were either bilaterally or unilaterally injected into the targeted brain region using an electric microinjection pump (Stoelting, USA) at an infusion rate of 30–50 nl  min^−1^. The glass pipette remained at the injection site for 10–15 min after infusion and was then slowly removed to avoid viral backflow. Viral injection coordinates were: GR (anteroposterior (AP), −7.75 mm; mediolateral (ML), ±0.3 mm; dorsoventral (DV), −4.1 mm, relative to bregma), DRN (AP, −4.5 mm; ML, 0.25 mm; DV, −3.3 mm) and mPFC (AP, +1.80 mm; ML, ±0.35 mm; DV, −2.5 mm). AAV expression was administered for at least 2 weeks before the experiments. For fiber photometry recordings, optical fibers (200 µm; OD = 0.37 NA; Inper) were unilaterally implanted 200–300 µm above the virus injection sites. After implantation, optical fibers were secured to the skull with bone screws and dental cement. Individual animals failing to achieve an AAV‐mediated gene transfer into the target region were excluded from the experiments.

### Viruses

To label neurons in downstream targets, AAV2/9‐CAG‐EGFP‐WPRE (titer: 1.05 × 10^12^  vector genomes (vg) ml^−1^) was injected into the GR of C57BL/6 mice.

To label neurons in the upstream nucleus, AAV2/Retro‐CAG‐mCherry‐WPRE (titer: 2.15 ×  10^13^ vg ml^−1^) was injected into the DRN of C57BL/6 mice. Red retrobeads (Lumafluor, US) were injected into the GR, to label DRG neurons.

To specifically track GR‐projecting neurons in the DRN, AAV2/1‐hSyn‐Cre (titer: 2.08 × 10^12^ vg ml^−1^) was injected into the GR and AAV2/9‐hSyn‐DIO‐mCherry (titer: 1.25 × 10^12^ vg ml^−1^) into the DRN. Three weeks later, mice were euthanized and perfused for immunostaining.

To retrograde track GR neurons projecting to the DRN, AAV2/Retro‐hSyn‐Cre (titer: 1.03 × 10^13^ vg ml^−1^) was injected into the DRN and AAV2/9‐hSyn‐DIO‐mCherry (titer: 1.25 × 10^12^ vg ml^−1^) into the GR. Three weeks later, mice were euthanized and perfused for immunostaining.

To identify projection targets of DRN postsynaptic neurons, AAV2/1‐hSyn‐Cre (titer: 2.36 × 10^13^ vg ml^−1^) was injected into the GR and AAV2/9‐hSyn‐DIO‐mGFP‐T2A‐Synaptophysin‐mRuby (titer: 3.25 × 10^12^ vg ml^−1^) into DRN.

For chemogenetic inhibition of the GR‐DRN‐mPFC pathway, AAV2/1‐hSyn‐Cre (titer: 2.36 × 10^13^ vg ml^−1^) was injected into the GR and AAV2/9‐hSyn‐DIO‐hM4Di‐mCherry (titer: 2.6 × 10^12^ vg ml^−1^) into DRN. AAV2/1‐hSyn‐Cre (titer: 2.36 × 10^13^ vg ml^−1^) was injected into DRN and AAV2/9‐hSyn‐DIO‐hM4Di‐mCherry (titer: 2.6 × 10^12^ vg ml^−1^) into the mPFC. AAV2/Retro‐hSyn‐Cre (titer: 1.03 × 10^13^ vg ml^−1^) was injected into DRN and AAV2/9‐hSyn‐DIO‐hM4Di‐mCherry (titer: 2.6 × 10^12^ vg ml^−1^) into the GR. Electrophysiological recordings and immunostaining were performed at 3 weeks after injections.

For chemogenetic activation of the GR‐DRN‐mPFC pathway, AAV2/1‐hSyn‐Cre (titer: 2.36 × 10^13^ vg ml^−1^) was injected into the GR and AAV2/9‐hSyn‐DIO‐hM3Dq‐mCherry (titer: 1.69 × 10^13^ vg ml^−1^) into DRN. AAV2/1‐hSyn‐Cre (titer: 2.36 × 10^13^ vg ml^−1^) was injected into DRN and AAV2/9‐hSyn‐DIO‐hM3Dq‐mCherry (titer: 1.69 × 10^13^ vg ml^−1^) into the mPFC. AAV2/Retro‐hSyn‐Cre (titer: 1.03 × 10^13^ vg ml^−1^) was injected into DRN and AAV2/9‐hSyn‐DIO‐hM3Dq‐mCherry (titer: 1.69 × 10^13^ vg ml^−1^) into the GR. Electrophysiological recordings and immunostaining were performed at 3 weeks after injections.

For fiber photometry recordings, AAV2/1‐hSyn‐Cre (titer: 2.08 × 10^12^ vg ml^−1^) was injected into the GR and AAV2/9‐hSyn‐DIO‐jGCaMP7s (titer: 5.08 × 10^12^ vg ml^−1^) into DRN. Three weeks later, an optical fiber (Inper) was implanted into DRN for photometry recordings.

For recordings of 5‐HT levels in the mPFC, AAV2/9‐hSyn‐g5HT3.0 (titer: 5.29 ×  10^12^ vg ml^−1^) licensed by Yulong Li's Lab (Peking University) was injected into the mPFC. Three weeks later, an optical fiber (Inper) was implanted into the mPFC for photometry recordings. All viruses were obtained from OBiO Technology (Shanghai, China) or BrainVTA (Wuhan, China).

### Chemogenetic Manipulation

For chemogenetic activation or inhibition, CNO (2 mg kg^−1^ in 0.1 ml, i.p; C0832, SigmaAldrich) was administered 30 min prior to restraint or social defeat stress or treadmill exercise.

### Fiber Photometry Recordings and Analysis

A multichannel optical fiber recording system (Inper) was used to record GCaMP and GRAB5‐HT2 h fluorescent intensities in the DRN or mPFC. AAV2/9‐hSyn‐DIO‐jGCaMP7s virus was injected into the GR and AAV2/9‐hSyn‐DIO‐jGCaMP7s virus into DRN, then optical fibers were implanted into DRN. To detect 5‐HT release, AAV2/9‐hSyn‐g5HT3.0 virus was injected into the mPFC. Treadmill exercises were then conducted at 3 weeks after virus infection. The fluorescence of Ca^2+^ and g5HT3.0 were recorded before and after exercising. A laser (470 and 410 nm) was used to detect jGCaMP7s or g5HT3.0 signals and autofluorescence. The 410 nm channel served as a control channel and was subtracted from the jGCaMP7s and g5HT3.0 channels to eliminate autofluorescence, bleaching and movement effects. The laser power at the tip of the optical fiber was adjusted to 20–40 µW. An Inper Data Process was used to analyze photometric data, draw heat maps and average Ca2+ traces. The baseline was defined as the average Ca2+ instantaneous 2s before onset of the event. The Z score (Δ F / F) of the fluorescent change and the area under the curve of the transient Ca^2+^ change corresponding to the event were analyzed. After recording, mice were perfused with 4% paraformaldehyde (PFA) and assayed to confirm virus expression and verify fiber placement. Data from mice satisfying these criteria were then used in the analyses.

### In Vitro Electrophysiological Recordings

Three weeks after virus injection, mice were deeply anesthetized, the entire brain was rapidly removed and immersed in a pre‐oxygenated (95% O2, 5% CO2, v/v) cold cutting solution (119 mm choline chloride, 26 mm NaHCO3, 30 mm glucose, 2.5 mm KCl, 7 mm MgSO4, 3 mm sodiumpyruvate, 1 mm NaH2PO4, 1 mm CaCl2, 1.3 mm sodium l‐ascorbate and 1 mm kynurenicacid). Sections (300 µm) containing DRN or mPFC were cut using a vibratome (VT1200S, Leica, Germany). Sections were transferred to a recovery solution (85 mm NaCl, 50 mm sucrose, 24 mm NaHCO3, 25 mm glucose, 4 mm MgCl2, 2.5 mm KCl, 1.25 mm NaH2PO4 and 0.5 mm CaCl2) saturated with 95% O2 and 5% CO2, incubated for 30 min at 37 °C and then at room temperature for another 30 min before recordings.

After incubation, sections were placed in the recording chamber with ACSF (130 mm CsMeSO4, 10 mm CsCl, 10 mm Hepes, 10 mm phosphocreatine, 5 mm Mg‐ATP (adenosine 5′‐triphosphate), 5 mm EGTA, 4 mm NaCl, 1 mm MgCl2, 0.5 mm Na3‐GTP (guanosine 5′‐triphosphate) and 4 mM QX‐314 with a pH of 7.35). A glass pipette (4 to 6 megohms) was prepared and whole‐cell recordings were performed on a MultiClamp 700B amplifier. Under an upright fluorescent microscope, brain sections of the DRN or mPFC were observed with a 60x water‐immersion lens. For sEPSC recordings, electrodes were filled with 115 mm CsMeSO3, 20 mm CsCl, 10 mm Hepes, 10 mm Na‐phosphocreatine, 2.5 mm MgCl2, 4 mm Na2‐ATP, 0.4 mm Na‐GTP, and 0.6 mm EGTA, with the the neurons then being placed under −70 mV to record sEPSCs. The data were filtered at 2 kHz and sampled at 10 kHz using Digidata 1440A. For CNO treatment, neurons were rested for 5 min after applying currents in 20 pA steps (from −60 to 240 pA). Slices were then perfused with ACSF containing 5 µm CNO. At 10 min after CNO infusion, the same current‐clamp procedure as described above was performed. Data were analyzed using the Mini Analysis Program (Synaptosoft) and Clmapfit 10.6 software (Molecular Devices).

### Immunochemistry

Mice were deeply anesthetized, transcardially perfused with 0.9% normal saline and immobilized with 4% PFA. Brains or DRG were removed, postfixed in 4% PFA overnight at 4 °C and dehydrated in PBS with a sucrose gradient (10 to 30%) at 4 °C. Brains (30 µm) and DRG (8 µm) were sectioned on a cryostat microtome (CM1860, Leica, Germany) and incubated with blocking buffer for 3 h at room temperature and overnight at 4 °C with primary antibodies.

The primary antibodies used were: c‐Fos (1:3000; CST), CAMKII‐α (1:200, Santa Cruz), GAD‐67 (1:200, Santa Cruz), TPH2 (1:200, Bioworld), PSD95 (1:200; CST), VMAT2 (1:200, Santa Cruz), and Vglut1 (1:200, Bioworld). The sections were subsequently washed three times in PBS and incubated at 37 °C for 1 h using corresponding Alexa Fluor 488/568/Cyanine 5, with conjugated secondary antibodies (1:500, Invitrogen). DAPI (Beyotime) used for nuclear staining. All images were captured with an Olympus VS120 high‐throughput fluorescent or confocal microscope (LSM880, Carl Zeiss, Germany).

### Imaging and Analysis

Three‐six C57BL/6 mice were used to quantify the cell types of postsynaptic neurons in GR, DRN and mPFC brain regions for each experiment. At three weeks after AAV infection, the number of mCherry‐labelled neurons and double‐labeled neurons with CaMKII‐α, GAD67, and TPH2 were counted in three consecutive brain sections (30 µm per section) within the brain target regions of each mouse. The percent of double‐labelled neurons was calculated as the percent of the total number of double‐labeled neurons counted in 6 mice within the total number of mCherry‐labeled neurons counted in 6 mice.

To quantify the influence of acute treadmill exercise on c‐Fos expression in the GR, DRN, mPFC, hippcampus, LHb, amygdala, and DRG, 16 mice were equally divided into two groups, the sedentary group and the treadmill‐exercise group. In each mouse, the number of c‐Fos^+^ cells was counted within three consecutive sections (30 µm per brain section, 8 µm per DRG section) of GR, DRN, mPFC, hippocampus, LHb, amygdala and DRG. The average number of c‐Fos^+^ cells in the brain section and DRG was calculated as the total number of c‐Fos+ cells counted in 8 animals divided by the number of animals.

To quantify the effect of chemogenetic inhibition or activation on c‐Fos expression, all animals in the experimental and control groups were respectively injected with either saline or CNO, and all were anesthetized and perfused after 30 min. The corresponding brain regions (GR, DRN, mPFC) were immunostained with c‐Fos. For chemogenetic activation, the number of hM3Dq‐labeled neurons and c‐Fos/hM3Dq‐double‐labeled neurons were counted in three consecutive brain slices per mouse. The percent of c‐Fos/hM3Dq double‐labeled neurons in each group was calculated as a percent of the total number of c‐Fos/hM3Dq double‐labeled neurons counted in 6 mice as divided by the total number of hM3Dq‐labeled neurons counted in 6 mice. For chemical inhibition, the number of hM4Di‐labeled neurons and c‐Fos/hM4Di‐double‐labeled neurons were counted in three consecutive brain slices per mouse.

### Western Blotting

Mouse brain tissues were homogenized in cold RIPA Buffer supplemented with protease and phosphatase inhibitors. The supernatants of tissue lysates were aspirated and centrifuged (20 min, 12 000 rpm, 4 °C). Equal amounts of proteins were separated by SDS‐PAGE, transferred to PVDF membranes, with the transfer membranes carrying the target proteins then incubated in 5% BSA diluted in 1× Tris‐Buffered Saline (TBS)‐Tween 20 for 2 h. The membranes were incubated with primary antibodies overnight at 4 °C, then with horseradish peroxidase‐conjugates sheep anti‐mouse IgG or sheep anti‐rabbit IgG as the secondary antibody. Immunoblots were generated on the membrane with use of enhanced chemiluminescence (ECL) and Image‐J software was used to perform pixel quantifications of the images. The antibodies used were: anti‐PSD95 (1:1000; CST), anti‐Synaptophysin (1:1000; CST), anti‐BDNF (1:1000; Affinity), anti‐CREB (1:1000; CST), anti‐Phospho‐CREB (1:1000; CST), anti‐CaMKII (1:1000; CST), anti‐Phospho‐CaMKII (1:1000; CST), anti‐TPH2 (1:1000, Bioworld), and anti‐VMAT2(1:1000, Santa Cruz).

### Behavioral Assays

Before all behavioral tests, mice were transported to the experimental room to acclimate for 2 h. With the exception of the social interaction tests, all behavioral boxes were wiped with 75% ethanol and air‐dried before each trial. All behavioral experiments were conducted in isolated behavioral testing rooms and operators were blinded as to the experimental treatment group.

### Social Interaction Test (SI)

The SI test was performed in an open arena (50 × 50 cm) to observe the social behavioral after CSDS. During the initial 2.5 min non‐social stimulus phase period, mice were allowed to freely explore the open arena. Amount of time in the area around the wired mesh cage (interaction zone) and in the area along the wall on the opposite side of the cage (oppositional zone) were recorded. An unfamiliar CD1 mouse was then placed in the wire cage and during this 2.5 min social‐stimulus phase period, mice were again free to explore the arena. Calculation of the SI ratio consisted of dividing the amount of time spent with the social stimulus by the time spent with the non‐social stimulus.

### Sucrose Preference Test (SPT)

The SPT was performed as previously described with minor modifications.^[^ ^76^, ^77^ ^]^ Briefly, mice were placed individually in cages with two bottles of 2% sucrose solution for the first 24 h, with the sucrose solution then being replaced with water for the next 24 h. Following an acclimation period, mice were deprived water for 12 h, after which they were allowed free access to two bottles for 6 h, one containing a 2% sucrose solution and one containing water. Sucrose preference was defined as sucrose consumption / [water consumption + sucrose consumption] × 100% during the 6 h test.

### Forced Swimming Test (FST)

The forced swimming test was performed under normal light as described previously.^[^ ^76^ ^]^ Mice were placed in a cylinder of water (temperature 23–25 °C; diameter 20 cm, height 25 cm) for 6 min. Depth of the water was set to prevent the animal from touching the bottom with their hind limbs. Immobility times were calculated, with immobility being defined as floating or remaining motionless with no active movement except to prevent drowning.

### Open Field Test (OFT)

Motor activity and anxiety were assessed using an open field arena (50 × 50 × 40 cm). Mice were placed in the center of the arena with dim lighting and allowed to explore for 10 min. An infrared camera positioned above the box recorded all animal movements. Total spontaneous activity and time spent in this central area were analyzed offline using Topscan software.

### Elevated Plus Maze Test (EPM)

The elevated plus maze used in this study consisted of two open arms (35 × 6 cm), two closed arms (35 × 6 × 20 cm), and a central platform area (6 × 6 cm). The maze was positioned at a height of 50 cm above the floor in a room with standard lighting conditions. Each mouse was individually placed on the central platform and allowed to freely explore the maze for 6 min. The video tracking system, Top scan and its accompanying software, were employed for recording and analyzing the movement data.

### Statistics

All mice were randomly divided into control and experimental groups. All data were collected and processed randomly or in a counterbalanced manner. Experimenters were unaware of the treatments and all data analyses were performed blindly. Figure legends contain the number of subjects and the number of repetitions independently processed for each experimental condition. Animals that were incorrectly injected with virus or optical fibers were excluded from the analysis. Statistical methods were not used to predetermine sample sizes, with sample sizes in these experiments being similar to those as reported in previous publications. All data were presented as Means ± SEMs. The normality of the data was tested using the Shapiro–Wilk test, the paired Student's *t*‐test for evoked action potential statistics, and the two‐tailed unpaired Student's *t*‐test to evaluate the data from both groups. One way ANOVA with a Bonferroni post hoc test were used to evaluate data from more than two groups. A P value of < 0.05 was required for results to be considered as statistically significant. Statistical analyses and graph generation were performed using GraphPad Prism 10.0 and details regarding statistical analyses were contained in the figure legends.

## Conflict of Interest

The authors declare no conflict of interest.

## Author Contributions

S.Y. conceived the idea and designed the study. S.Y. and S.C. acquired the funding and supervised the study. T.L., Y.L., X.C., and W.W. performed the experiments. C.W. and H.L. performed statistical analysis. S.Y., T.L., and S.C. wrote and revised the manuscript. All authors read and approved the manuscript prior to submission.

## Supporting information



Supporting Information

Supplemental Data 1

## Data Availability

The data that support the findings of this study are available from the corresponding author upon reasonable request.
