# Supplementary material for: Exercise‐Activated mPFC Tri‐Synaptic Pathway Ameliorates Depression‐Like Behaviors in Mouse
Source: Adv Sci (Weinh). 2024 Nov 22;12(3):2408618. doi: 10.1002/advs.202408618 (PMC11744721; doi:10.1002/advs.202408618)
Supplement: Supplementary file 1 — Supporting Information [file ADVS-12-2408618-s002.docx]

**Exercise-activated mPFC tri-synaptic pathway ameliorates depression-like behaviors in mouse**

Tian Lan ^1,2^, Ye Li ^1,2^, Xiao Chen ^1,2^, Wenjing Wang ^1,2^, Changmin Wang ^1,2^, Haiyan Lou ^1,3^, Shihong Chen^4#^, Shuyan Yu ^1,2,5#^

1. Shandong Key Laboratory of Mental Disorders and Intelligent Control, The Second Hospital of Shandong University, School of Basic Medical Sciences, Shandong University, Jinan, Shandong, 250012, China.

2. Department of Physiology, School of Basic Medical Sciences, Cheeloo College of Medicine, Shandong University, Jinan, Shandong, 250012, China.

3. Department of Pharmacology, School of Basic Medical Sciences, Cheeloo College of Medicine, Shandong University, Jinan, Shandong, 250012, China.

4. Department of Endocrinology and Metabolism, The Second Hospital of Shandong University, Jinan, Shandong, China

5. Department of Medical Psychology and Ethics, School of Basic Medical sciences, Cheeloo College of Medicine, Shandong University, Jinan, Shandong, 250012, China;

﹡ Corresponding author: Shuyan Yu,

E-mail address: shuyanyu@sdu.edu.cn

Tel: +86-0531-88383902; fax: +86-0531-88382502


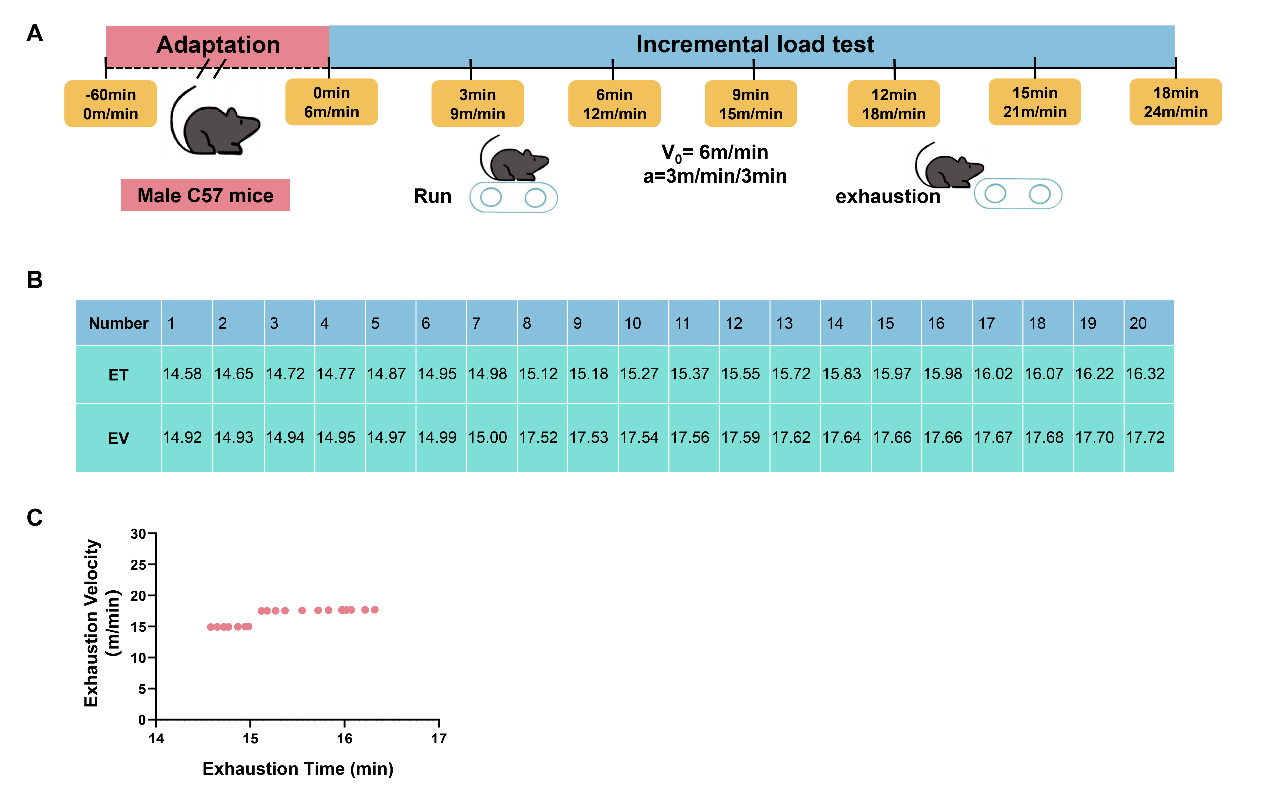


**Figure S1.** An optimal aerobic training velocity for treadmill exercise in mice. (A) Schematic of the incremental load test (ILT) for treadmill exercise in mice. (B) The exhaustion time (ET) and exhaustion velocity (EV) of each mouse. (C) Correlation of exhaustion time and exhaustion velocity of each mouse.


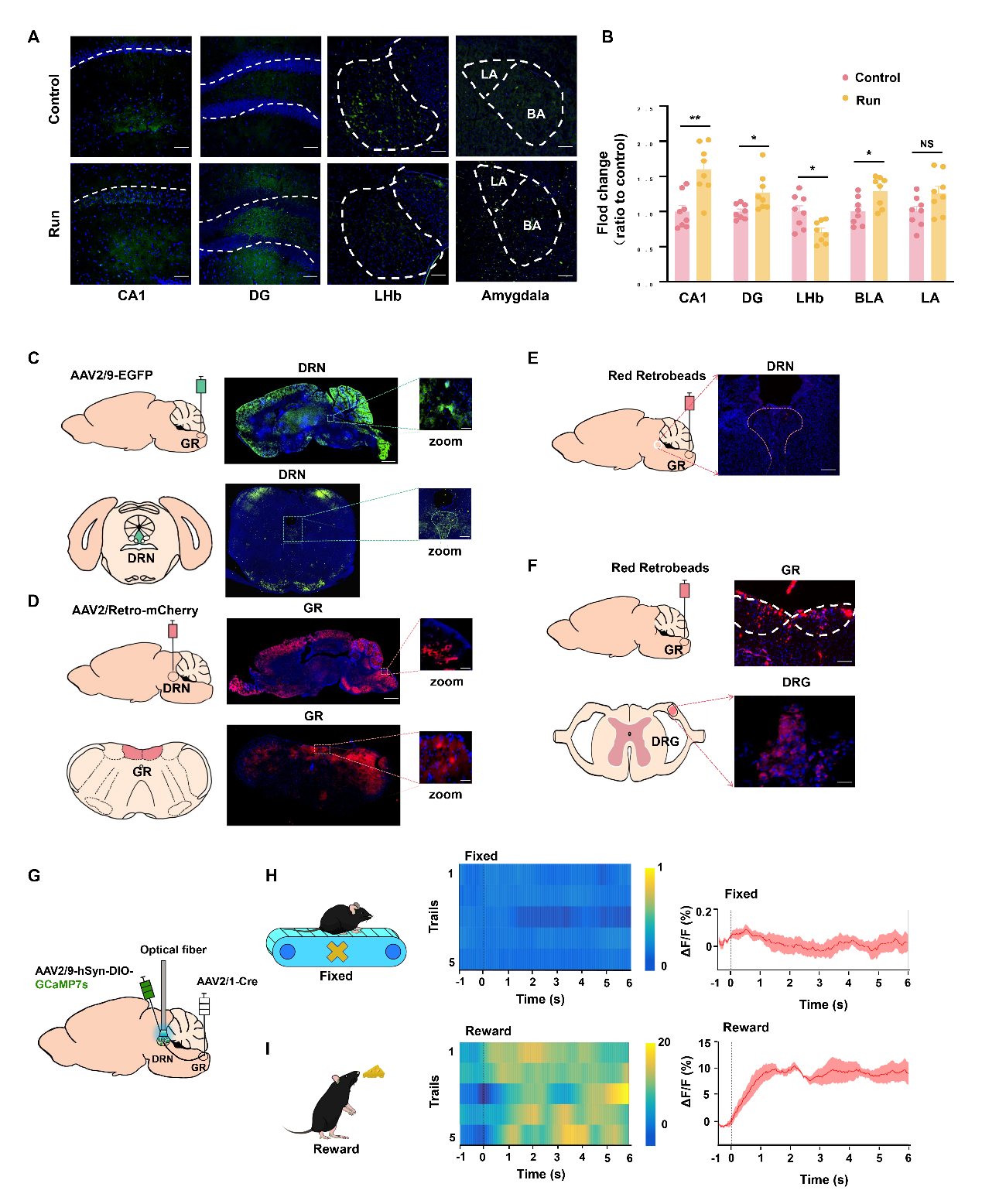


**Figure S2.** Anterograde and retrograde tracings and fiber recording of GR-DRN. (A) Typical image of Fos expression in CA1, LHb, amygdala and hippocampus. Scale bar: 50 μm. (B) Quantification of c-Fos^+^ cell numbers in the control and running mice (n = 8 mice/group). (C) Anterograde tracing and typical image of axonal terminals in DRN from the GR. Top: sagittal brain section. Scale bar: 1mm. Zoomed image: 100 μm. Bottom: coronal brain section. Scale bar: 500 μm. Zoomed image: 100 μm. (D) DRN retrograde tracing, representative image of mCherry-labeled neurons in the GR. Top: sagittal brain section. Scale bar: 1mm. Zoomed image: 100 μm. Bottom: coronal brain section. Scale bar: 200 μm. Zoomed image: 50 μm. (E) GR retrograde tracing, representative image of red retro-beads in the DRN. Scale bar: 100 μm. (F) GR retrograde tracing, representative image of red retro-beads in the DRG. Scale bar: 50 μm. (G) Schematic for monitoring calcium activities of GR-projecting DRN. (H-I) Heatmap and quantifications of calcium signal changes of GR-projecting DRN, as aligned to the onset (time = 0 s) of placing on the fixed treadmill (H) or giving a reward (I). N = 5 bouts from 5 mice. Two-tailed unpaired Student’s *t*-test for (E). *p < 0.05; **p < 0.01; NS, not significant. Data are presented as Means ± SEMs.


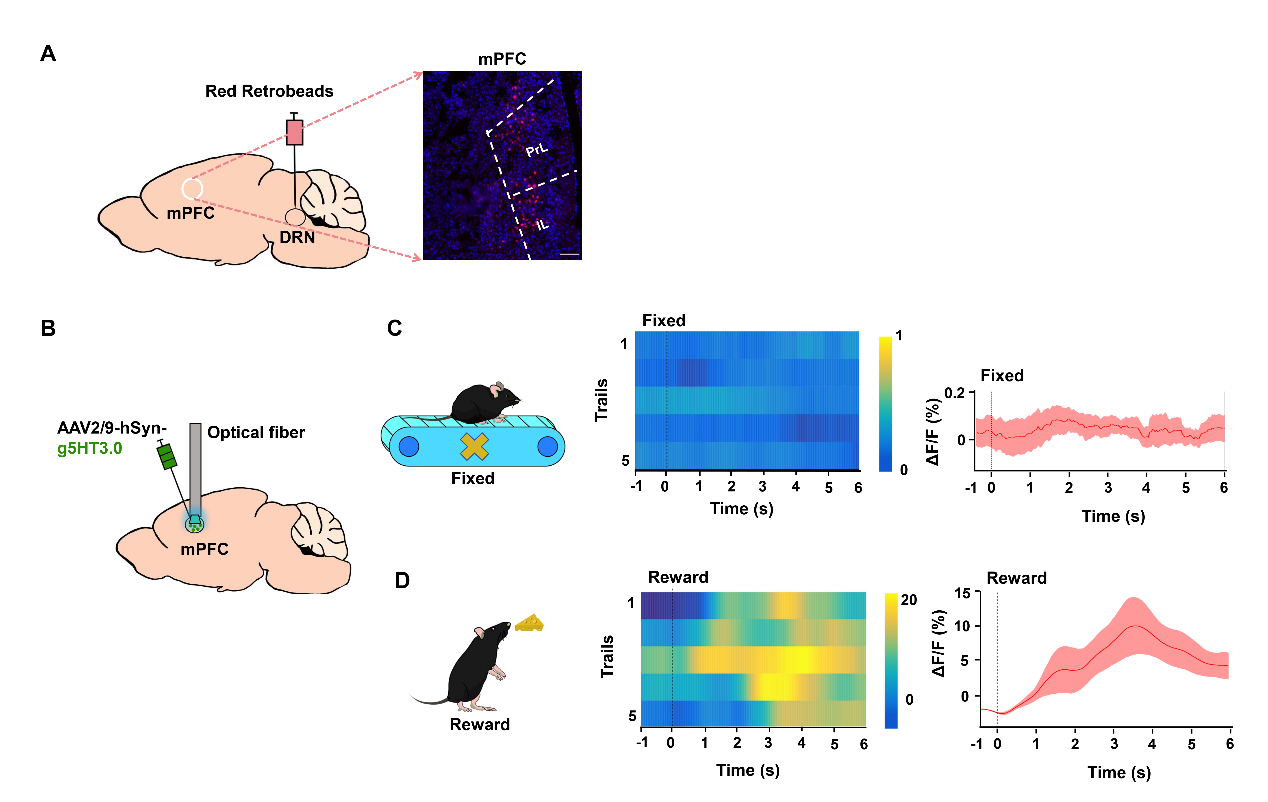


### Figure S3. Retrograde tracings DRN-mPFC and 5-HT release of mPFC in mice. (A) DRN retrograde tracing, representative image of red retro-beads in the mPFC. Scale bar: 100 μm. (B) Schematic for monitoring 5-HT release of mPFC. (C-D) Heatmap and quantifications of 5-HTrelease changes of mPFC, as aligned to the onset (time = 0 s) of placing on the fixed treadmill (C) or giving a reward (D). N = 5 bouts from 5 mice.


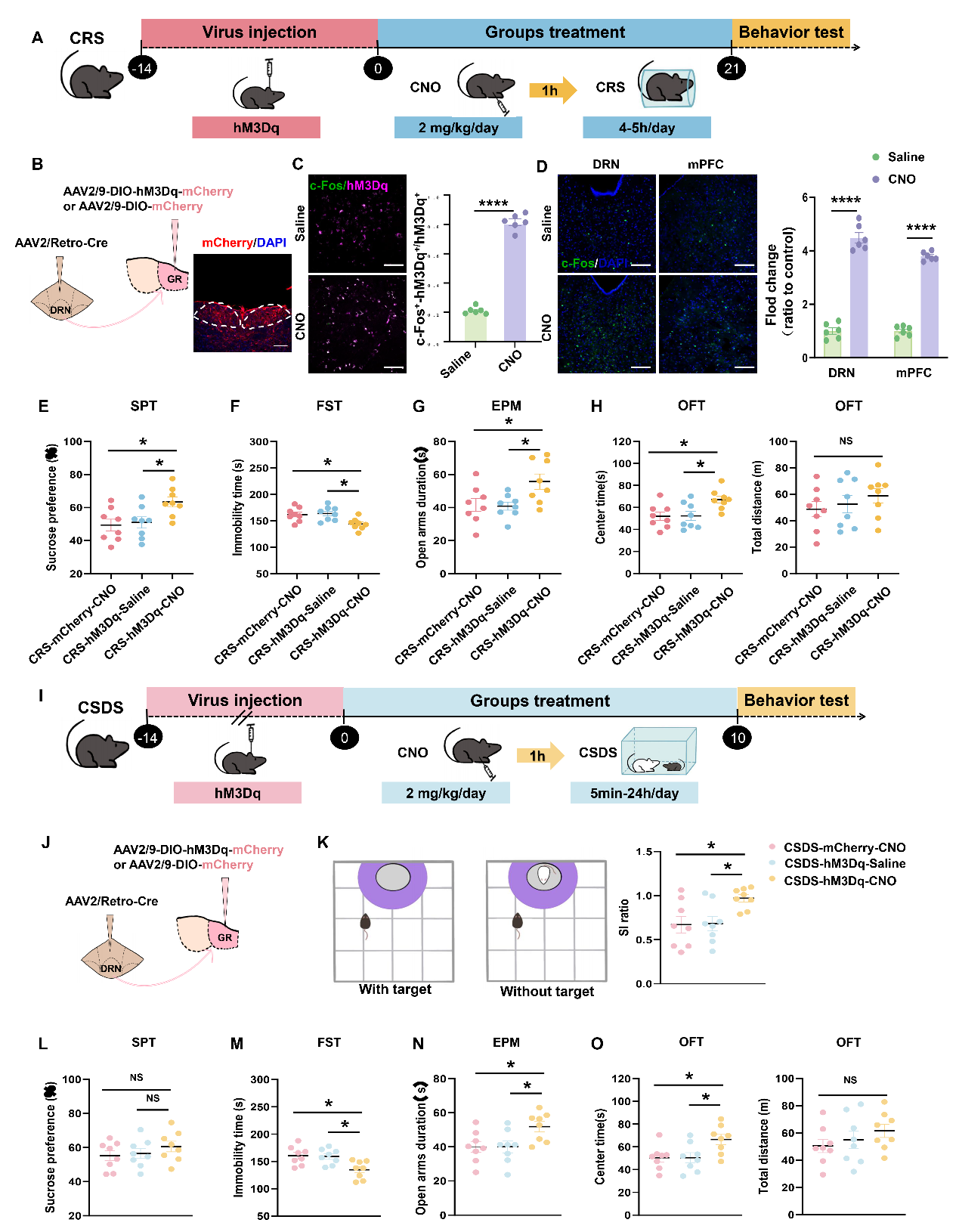


### Figure S4. Activation of the DRN-projecting GR neurons decreases depressive-like behaviors as induced by CRS and CSDS. (A) Schematic of the experimental design for the CRS model. (B) Schematic of virus injection and typical images of mCherry expression in GR. Scale bar: 50 μm. (C) Representative images and quantification of the GR showing c-Fos expression in neurons expressing hM3Dq in response to an i.p. injection of saline or CNO. Scale bar: 20 μm (n = 6 mice for each group). D) Representative images and quantification of DRN and mPFC showing c-Fos expression in response to an i.p. injection of saline or CNO. Scale bar: 50 μm (n = 6 mice/group). (E-H) Stress-induced depression-like and anxiety-like behaviors in the different experimental groups of the CRS model (n = 8 animals/group). SPT (E), FST (F), EPM (G) and OFT (H). (I) Schematic of the experimental design for the CSDS model. (J) Schematic of virus injection. (K) Social interactions of the different experimental groups in the CSDS model. (L-O) Sstress-induced depression-likeand anxiety-like behaviors in the different experimental groups of the CSDS model (n = 8 animals/group). SPT (L), FST (M), EPM (N) and OFT (O). Two-tailed unpaired Student’s *t*-test for (C-D). For (E-H) and (K-O): One-way analysis of variance (ANOVA) with Bonferroni post-hoc test. *, P<0.05; ****, P<0.0001; NS, no significant difference. Data are presented as Means ± SEMs.


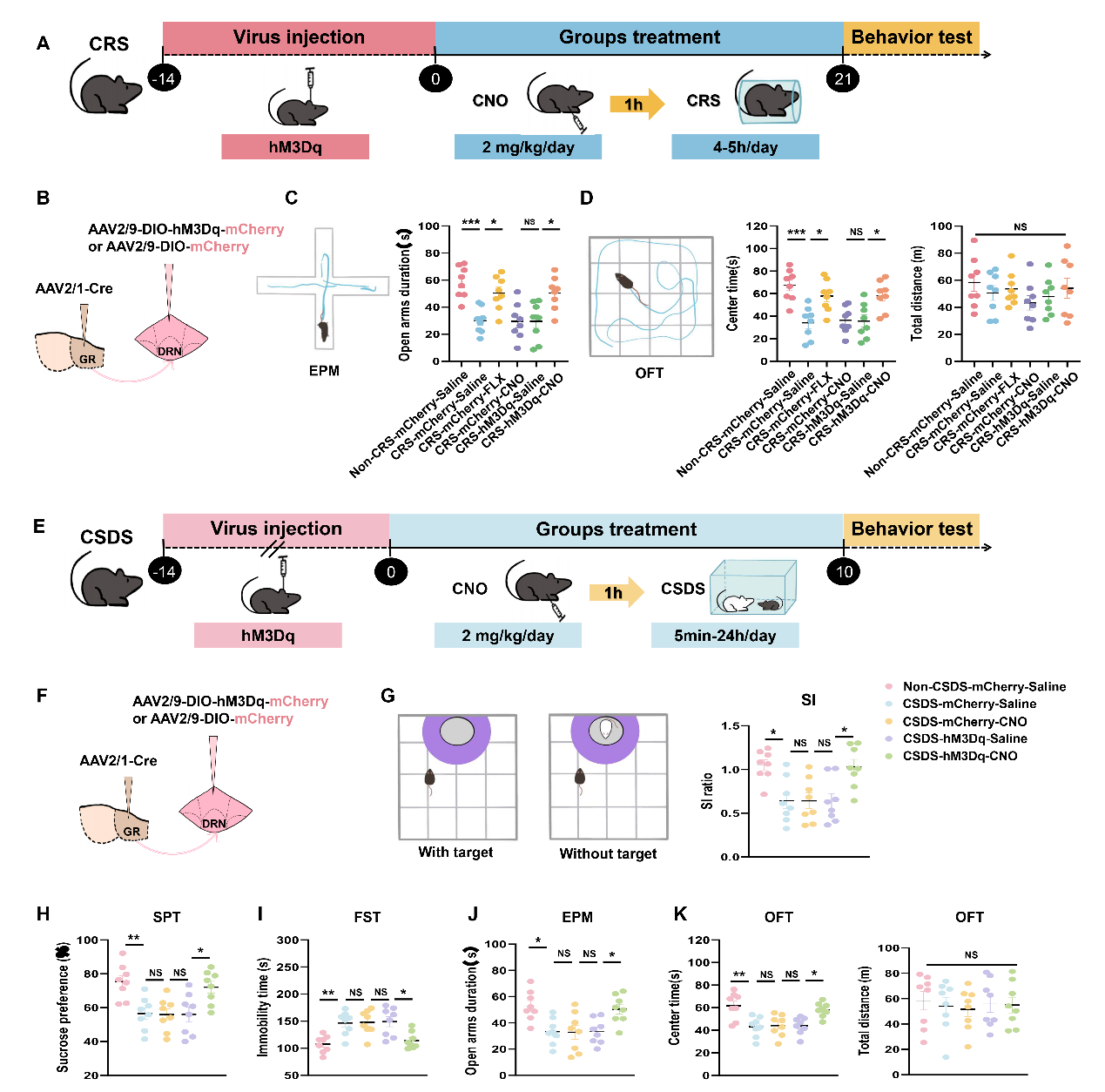


**Figure S5.** Activation of the GR-DRN pathway reverses depressive- and anxiety-like behaviors as induced by exposure to CRS and CSDS. (A) Schematic of the experimental design for the CRS model. (B) Schematic of virus injection. (C-D) Anxiety-like behaviors in the different experimental groups of the CRS model (n = 8 animals/group) in the EPM (C) and OFT (D). (E) Schematic of the experimental design for the CSDS model. (F) Schematic of virus injection. (G) Social interactions of the different experimental groups in the CSDS model. (H-K) Stress-induced depression-like and anxiety-like behaviors in the different experimental groups of the CSDS model (n = 8 animals/group), SPT (H), FST (I), EPM (J) and OFT (K). For all statistical tests: One-way analysis of variance (ANOVA) with Bonferroni post-hoc test. *, P<0.05; **, P<0.01; ***, P<0.001; NS, no significant difference. Data are presented as Means ± SEMs. Dots represent individual mice.


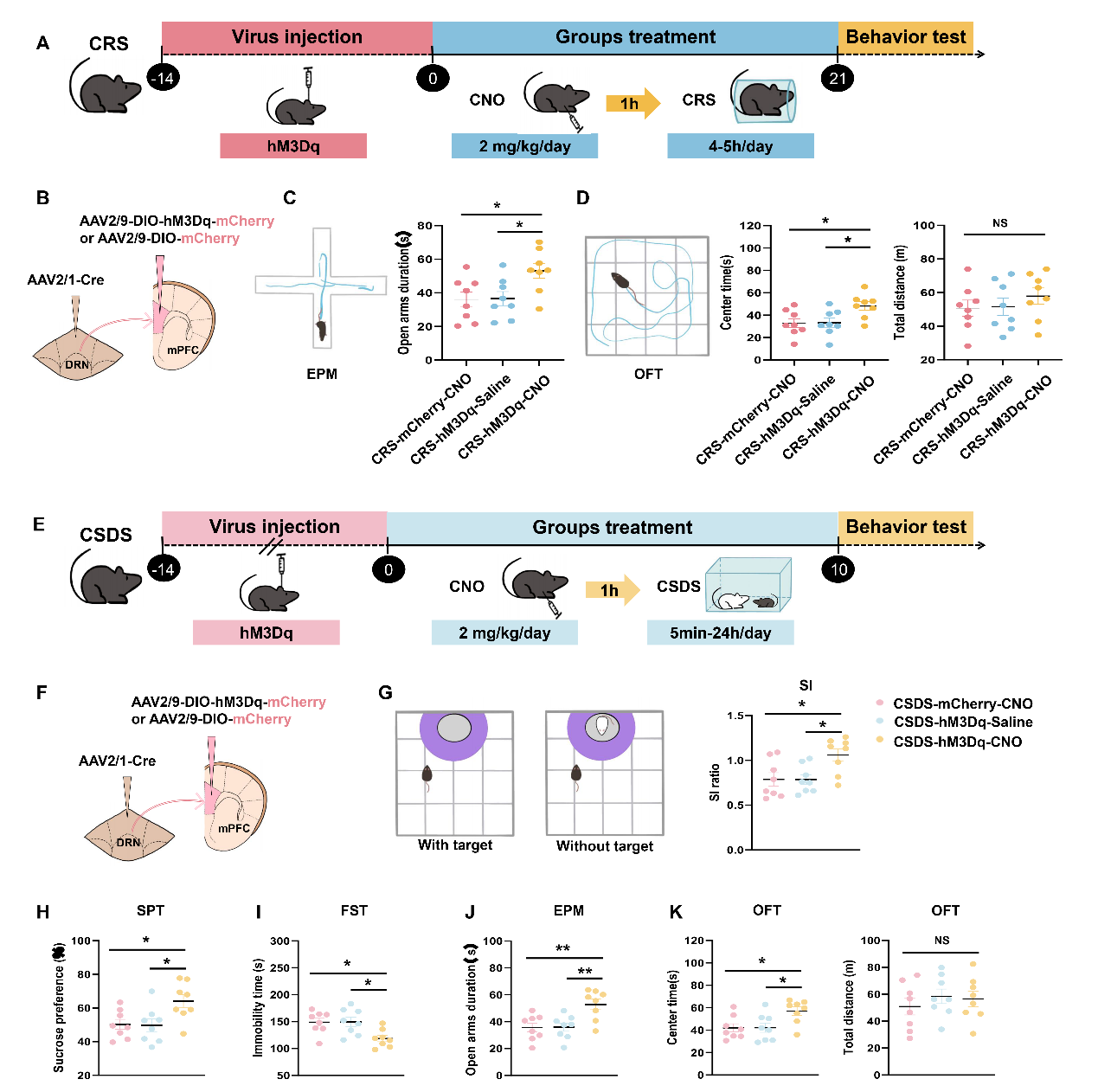


**Figure S6.** Activation of the DRN-mPFC pathway reverses depressive- and anxiety-like behaviors as induced by exposure to CRS and CSDS. (A) Schematic of the experimental design for the CRS model. (B) Schematic of virus injection. (C-D) Anxiety-like behaviors in the different experimental groups of the CRS model (n = 8 animals/group) in the EPM (C) and OFT (D). e Schematic of the experimental design for the CSDS model. (F) Schematic of virus injection. (G) Social interactions of the different experimental groups of the CSDS model. (H-K) Stress-induced depression-like and anxiety-like behaviors in the different experimental groups of the CSDS model (n = 8 animals/group), SPT (H), FST (I), EPM (J) and OFT (K). For all statistical tests: One-way analysis of variance (ANOVA) with Bonferroni post-hoc test. *, P<0.05; **, P<0.01; NS, no significant difference. Data are presented as Means ± SEMs. Dots represent individual mice.


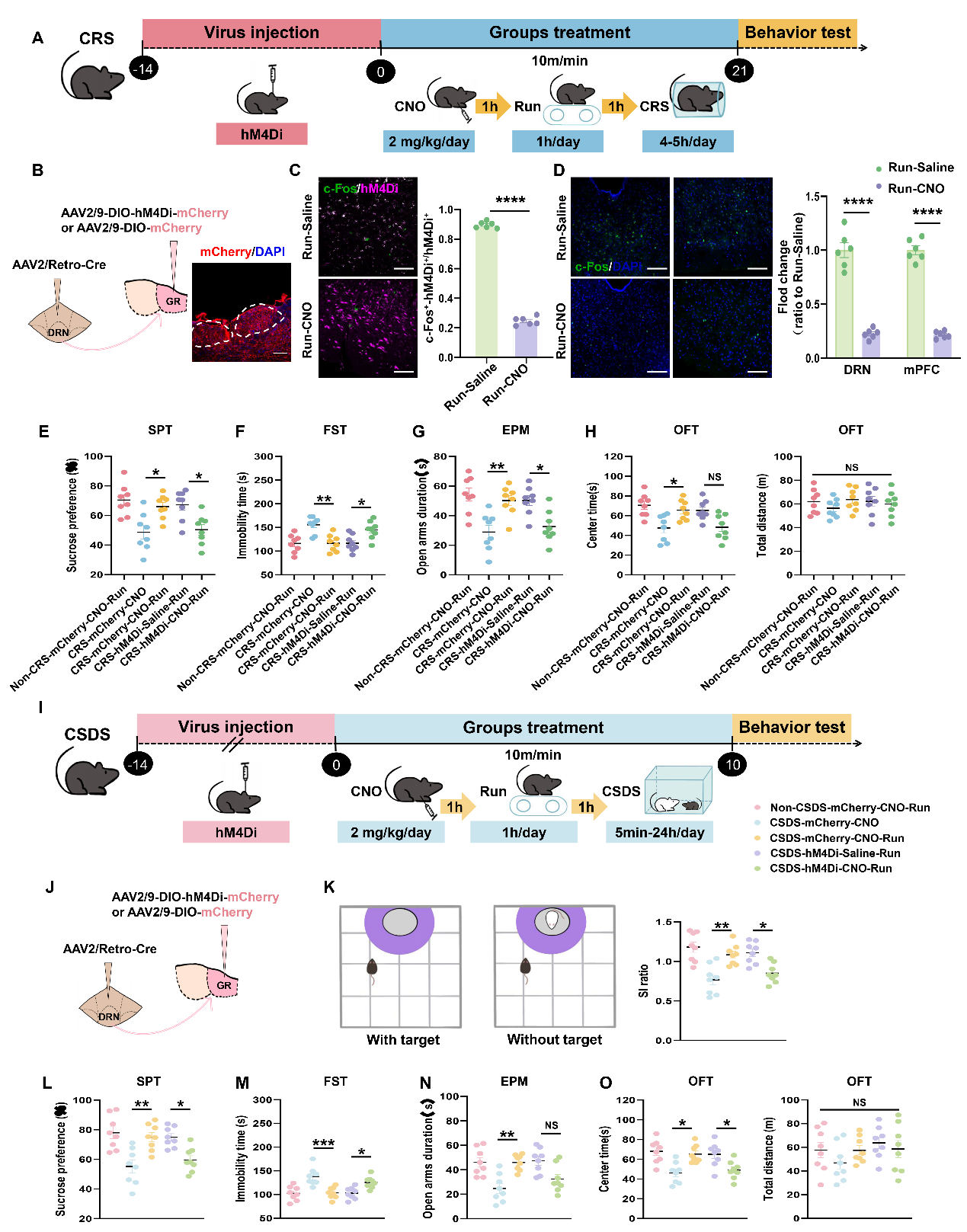


**Figure S7.** Inhibition of DRN-projecting GR neurons prevents exercise-induced antidepressant effects. (A) Schematic of experimental design for the CRS model. (B) Schematic of virus injection and typical images of mCherry expression in GR. Scale bar: 50 μm. (C) Representative images and quantification of the GR showing c-Fos expression in neurons expressing hM4Di in response to an i.p. injection of Run-saline or Run-CNO. Scale bar: 20 μm (n = 6 mice for each group). D) Representative images and quantification of DRN and mPFC showing c-Fos expression in response to an i.p. injection of saline or CNO. Scale bar: 50 μm (n = 6 mice/group). (E-H) Stress-induced depression-likeand anxiety-like behaviors in the different experimental groups of the CRS model (n = 8 animals/group). SPT (E), FST (F), EPM (G) and OFT (H). (I) Schematic of the experimental design for the CSDS model. (J) Schematic of virus injection. (K) Social interactions of the different experimental groups of the CSDS model. (L-O) Stress-induced depression-like and anxiety-like behaviors in the different experimental groups of the CSDS model (n = 8 animals/group). SPT (L), FST (M), EPM (N) and OFT (O). Two-tailed unpaired Student’s *t*-test for (C-D). For (E-H) and (L-O): One-way analysis of variance (ANOVA) with Bonferroni post-hoc test. *, P<0.05; **, P<0.01; ***, P<0.001; ****, P<0.0001; NS, no significant difference. Data are presented as Means ± SEMs.


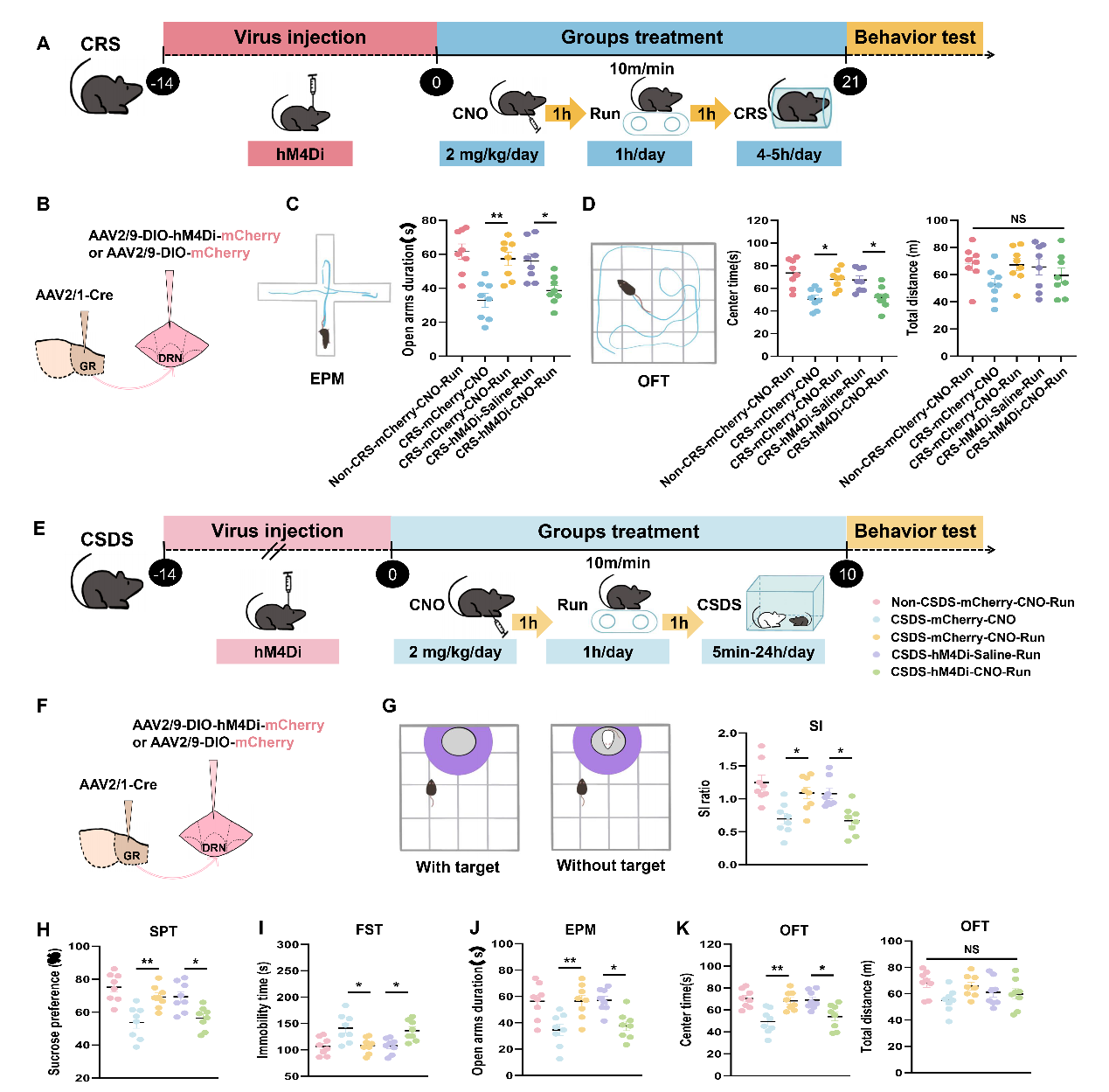


**Figure S8.** Inhibition of GR-DRN neurons prevents exercise-induced antidepressant effects. (A) Schematic of the experimental design for the CRS model. (B) Schematic of virus injection. (C-D) Anxiety-like behaviors in the different experimental groups of the CRS model (n = 8 animals/group) in the EPM (C) and OFT (D). (E) Schematic of the experimental design for the CSDS model. (F) Schematic of virus injection. (G) Social interactions of the different experimental groups of the CSDS model. (H-K) Stress-induced depression-like and anxiety-like behaviors in the different experimental groups of the CSDS model (n = 8 animals/group), SPT (H), FST (I), EPM (G) and OFT (K). For all statistical tests: One-way analysis of variance (ANOVA) with Bonferroni post-hoc test. *, P<0.05; **, P<0.01; NS, no significant difference. Data are presented as Means ± SEMs. Dots represent individual mice.


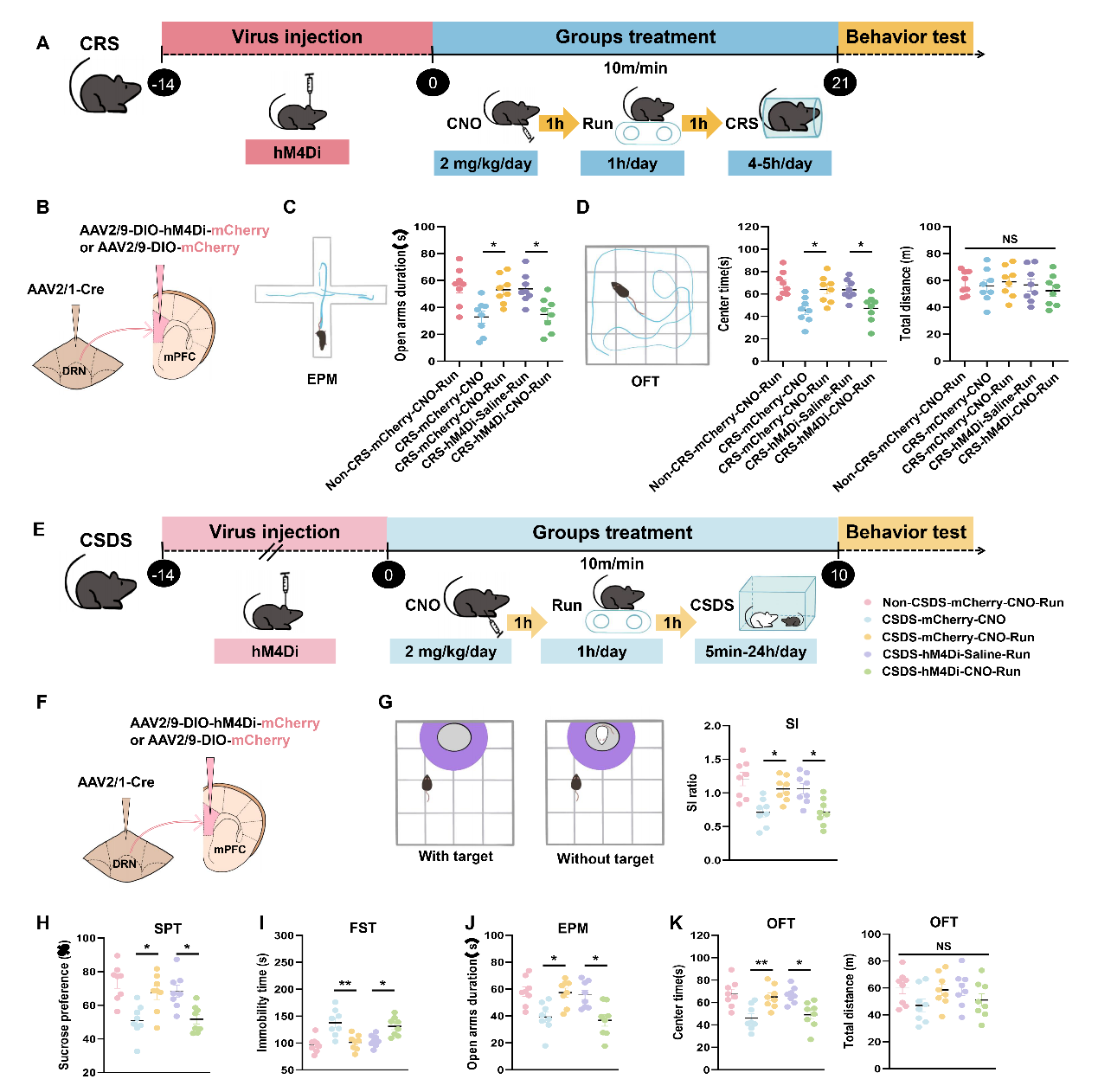


**Figure S9.** Inhibition of DRN-mPFC neurons prevents exercise-induced antidepressant effects. (A) Schematic of the experimental design for the CRS model. (B) Schematic of virus injection. (C-D) Anxiety-like behaviors in the different experimental groups of the CRS model (n = 8 animals/group) in the EPM (C) and OFT (D). e Schematic of the experimental design of the CSDS model. (F) Schematic of virus injection. (G) Social interactions of the different experimental groups of the CSDS model. (H-K) Stress-induced depression-like and anxiety-like behaviors in the different experimental groups of the CSDS model (n = 8 animals/group), SPT (H), FST (I), EPM (J) and OFT (K). For all statistical tests: One-way analysis of variance (ANOVA) with Bonferroni post-hoc test. *, P<0.05; **, P<0.01; NS, no significant difference. Data are presented as Means ± SEMs. Dots represent individual mice.


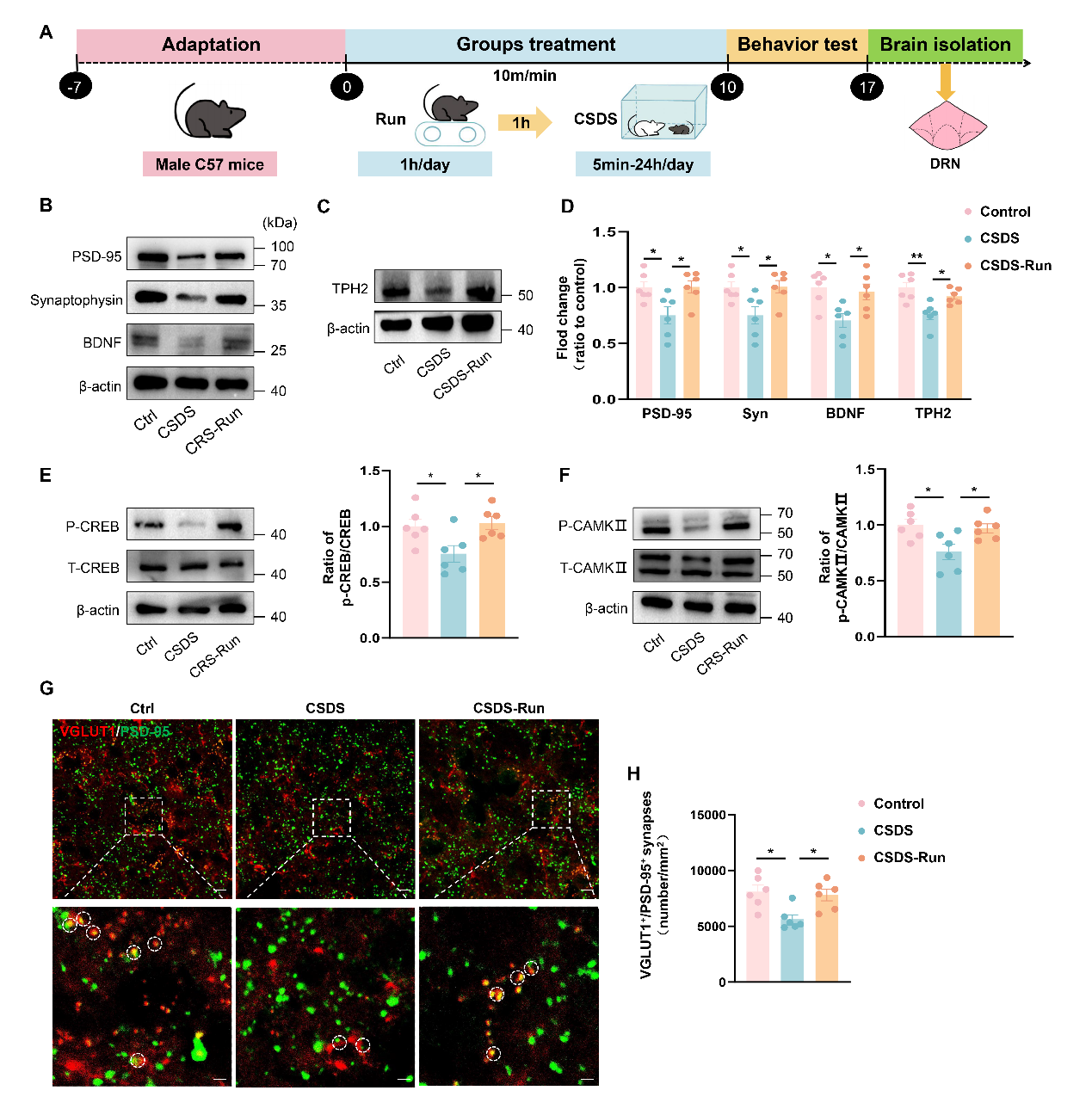


**Figure S10.** Exercise promotes neuronal synaptic plasticity of neurons within DRN. (A) Schematic of the experimental design for the CSDS model. (B-C) Representative western blots showing that exercise increased expression levels of synaptic structural proteins (B) and TPH2 (C) in DRN of mice exposed to CSDS. (D) Quantification of protein expression levels of PSD95, Synaptophysin, BDNF and TPH2 within the DRN region. (E-F) Representative western blots and quantification of p-CREB/CREB (E) and P-CAMKⅡ/CAMKⅡ (F) within the DRN region. (G) Immunofluorescent staining showing VGLUT1^+^ (red) and PSD95^+^ (green) co-localization in DRN neurons. Top: scale bar: 10 μm. Bottom: scale bar: 5 μm (n = 6 brain slices from 3 mice for each group). (H) The quantification of the synaptic number in DRN with each group. For (B-F): n = 6 animals/group. One-way analysis of variance (ANOVA) with Bonferroni post-hoc test. *, P<0.05; **, P<0.01; NS, no significant difference. Data are presented as Means ± SEMs.


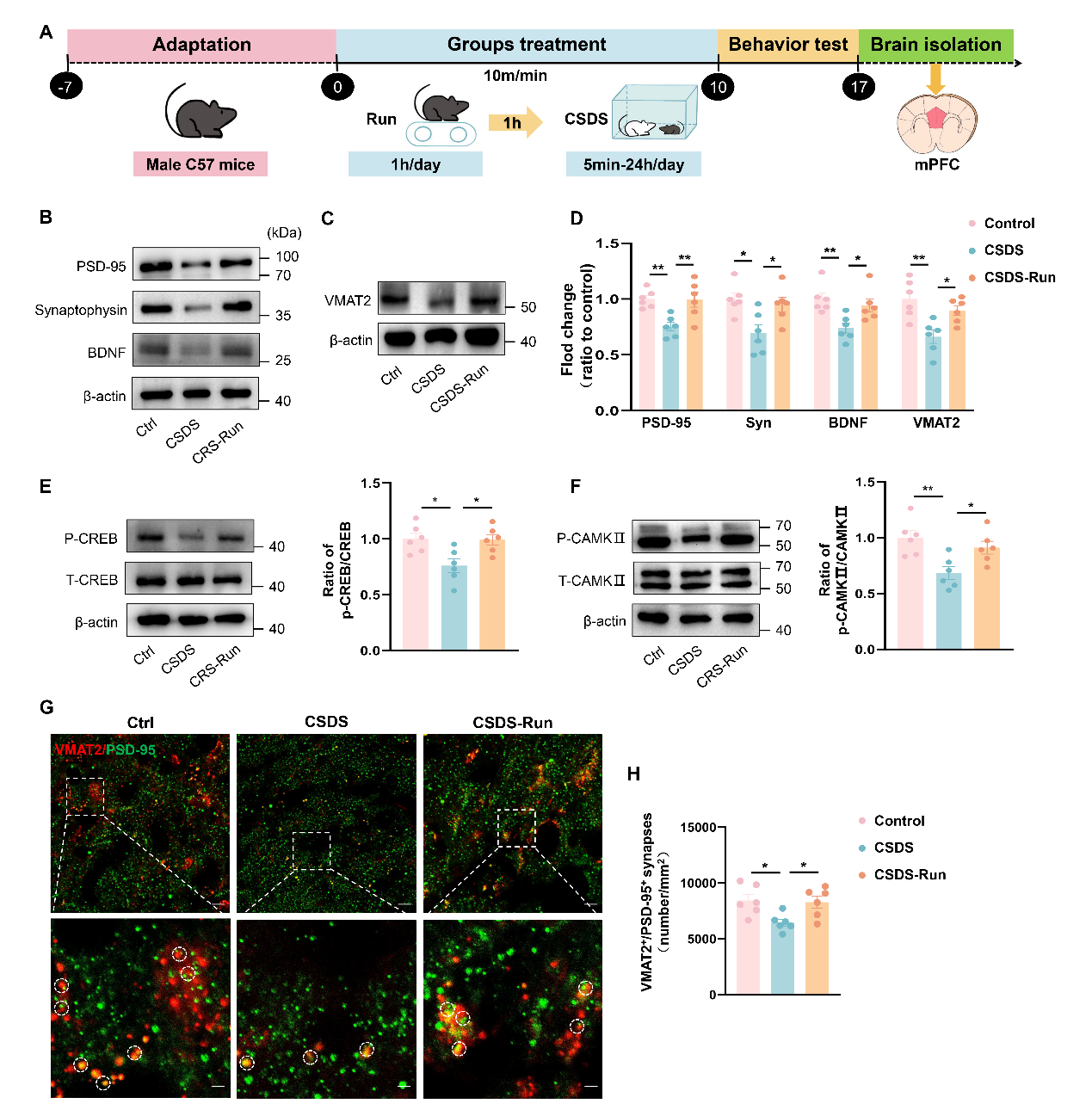


**Figure S11.** Exercise promotes neuronal synaptic plasticity of neurons within mPFC. (A) Schematic of the experimental design for the CSDS model. (B-C) Representative western blots showing that exercise increased the expression levels of synaptic structural proteins (B) and TPH2 (C) in the mPFC of mice exposed to CSDS. (D) Quantification of protein expression levels of PSD95, Synaptophysin, BDNF and TPH2 within the mPFC region. (E-F) Representative western blots and quantification of p-CREB/CREB (E) and P-CAMKⅡ/CAMKⅡ (F) within the mPFC region. (G) Immunofluorescent staining showing VMAT2^+^ (red) and PSD95^+^ (green) co-localization in DRN. Top: scale bar: 10 μm. Bottom: scale bar: 5 μm (n = 6 brain slices from 3 mice for each group). (H) The quantification of the synaptic number in mPFC with each group. For (B-F): n= 6 animals/group. One-way analysis of variance (ANOVA) with Bonferroni post-hoc test. *, P<0.05; **, P<0.01; NS, no significant difference. Data are presented as Means ± SEMs.


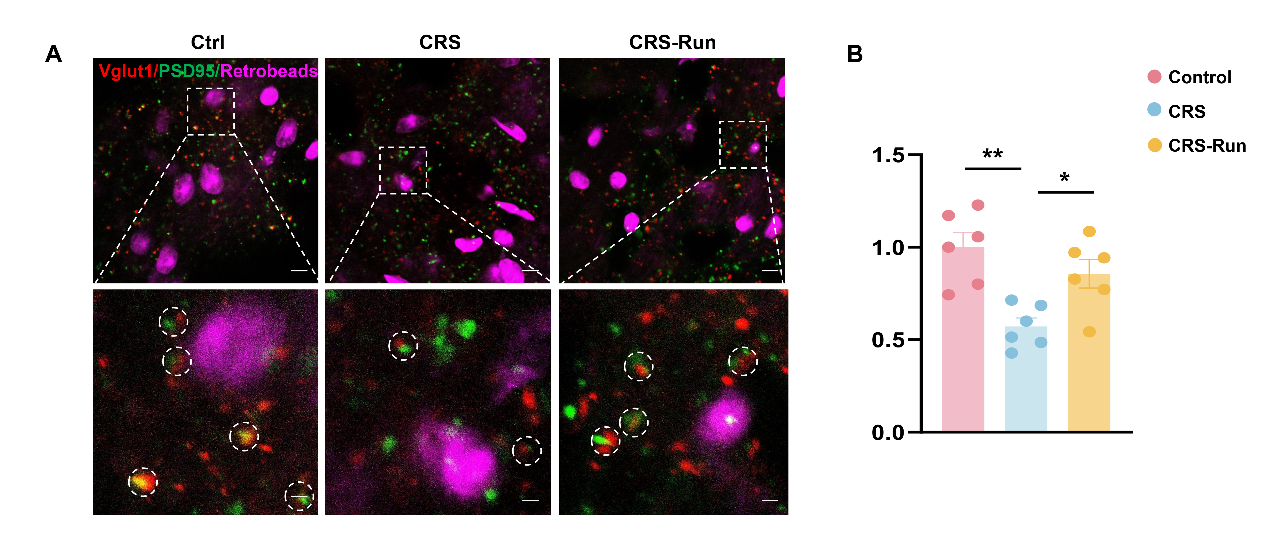


**Figure S12.** Exercise promotes neuronal synaptic plasticity of neurons in DRN projected to mPFC. (A) Immunofluorescent staining showing VGLUT1^+^ (red), PSD95^+^ (green) and RetroBeads (violet) co-localization in DRN. Top: scale bar: 10 μm. Bottom: scale bar: 5 μm (n = 6 brain slices from 3 mice for each group). (B) The quantification of the synaptic number in DRN with each group. One-way analysis of variance (ANOVA) with Bonferroni post-hoc test. *, P<0.05; **, P<0.01; NS, no significant difference. Data are presented as Means ± SEMs.
